# Redox-State-Driven Site Selectivity and Kinetic Gating in Polyoxovanadate Methylation

**DOI:** 10.1021/acs.inorgchem.6c01638

**Published:** 2026-08-06

**Authors:** Nghia Le, Pere Miró

**Affiliations:** Department of Chemistry, 4083 University of Iowa, Iowa City, Iowa 52240, United States

## Abstract

Polyoxovanadate clusters offer atomically defined platforms for studying how electronic structure influences reactivity at molecular metal-oxide surfaces. In this computational study, we investigated the coupling between reduction state and alkylation reactivity in the hexavanadate cluster [V_6_O_13_(TRIOL^X^)_2_]^2–^ (TRIOL = tris­(hydroxymethyl)­methane; X = −CH_3_, −NO_2_) through a systematic analysis of successive methylation and reduction pathways using density functional theory and microkinetic modeling. The calculations show that changes in electron count redistribute the nucleophilicity of bridging μ_2_-oxo ligands, leading to redox-dependent regioselectivity during sequential methylation. Microkinetic simulations further indicate that several thermodynamically accessible intermediates do not accumulate because they are rapidly consumed in subsequent transformations, consistent with the need for stepwise synthetic strategies to isolate certain dimethylated species. In addition, substituent effects on the TRIOL ligands follow Hammett-type correlations, allowing rapid estimation of electronic trends without additional quantum chemical calculations. Overall, the results provide a detailed picture of how reduction state influences accessibility and selectivity during polyoxovanadate methylation.

## Graphical abstract

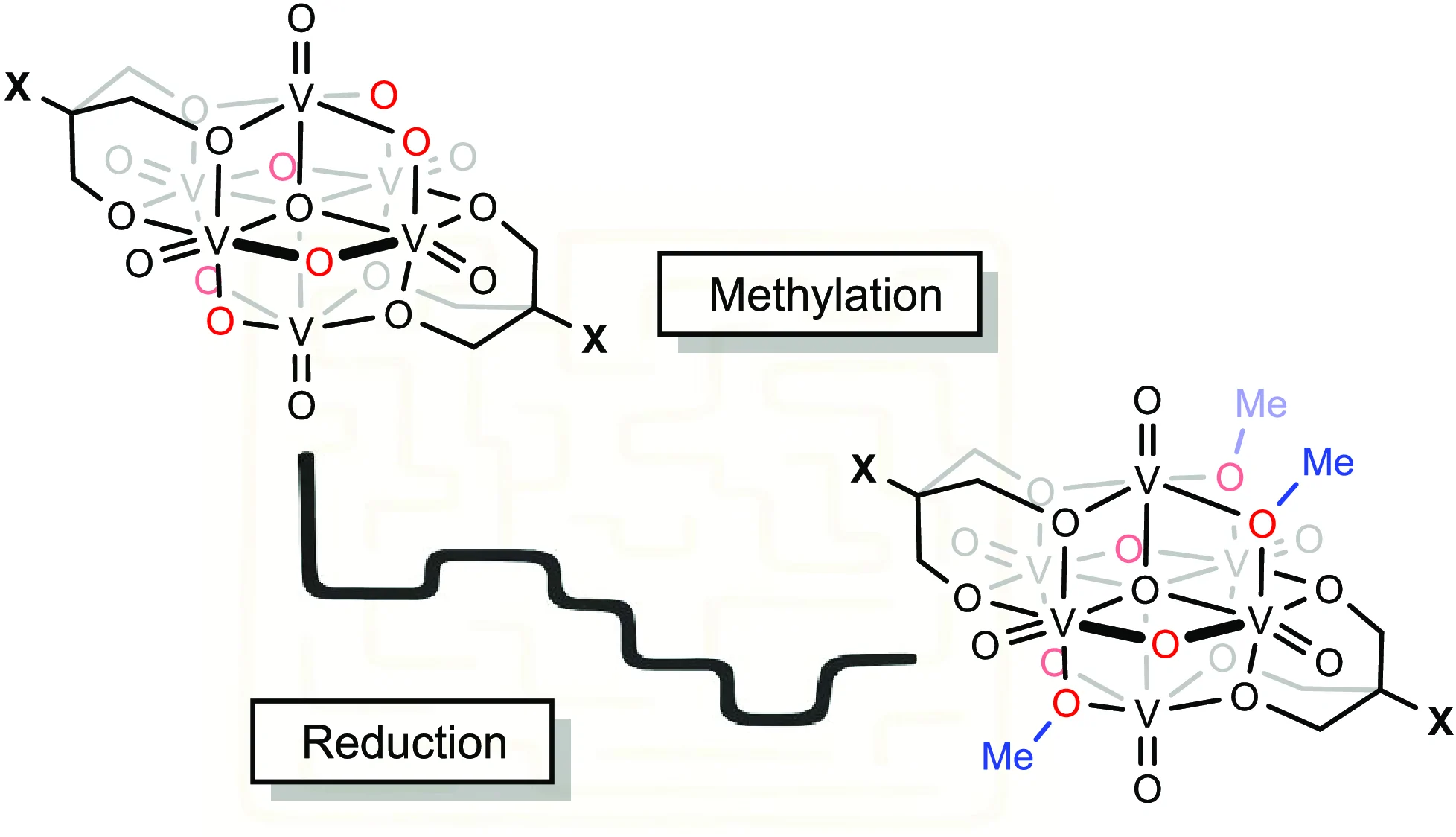

## Introduction

Polyoxovanadate clusters have emerged as valuable atomically precise molecular models that bridge the chemical understanding between discrete molecular metal oxides and extended bulk solids.^1–4^ These clusters combine a well-defined nuclearity with tunable electronic structure and surface composition, enabling detailed investigations into their nucleation and structure–function relationships that are often challenging to probe in extended oxide materials.^5–9^ Due to these attributes, polyoxovanadate clusters have been studied for diverse applications including material sciences,^10,11^ catalysis,^12–14^ and energy storage.^15–18^

The polyoxovanadate chemical space is mostly dominated by the decavanadate species in aqueous media; however, the hexameric Lindqvist-type core can be stabilized by various surface ligands during their synthesis.^19–22^ This approach allowed the systematic modulation of the electronic structure, solubility, and reactivity of these species while preserving the underlying molecular metal-oxide framework.^19,23^ An example is the incorporation of tris­(hydroxymethyl)­methane (TRIOL) ligands into an hexavanadate core, affording clusters of the general formula [V^V^_6_O_13_(TRIOL^X^)_2_]^2–^ (Figure 1, left). These polyoxovanadate frameworks have attracted attention due to their tunable redox properties and emerging biological activity, including their use as potential anticancer agents.^24^ In these systems, variation of the substituent on the TRIOL ligand provides a convenient handle for adjusting the cluster’s electronic properties, as reflected in systematic shifts of the redox potentials.^19^ Beyond redox behavior, changes in ligand identity also influence the protonation energetics and hydrogen atom transfer at the cluster surface, thereby impacting proton-coupled electron transfer processes associated with the molecular metal-oxide framework.^25^

**Figure 1:**
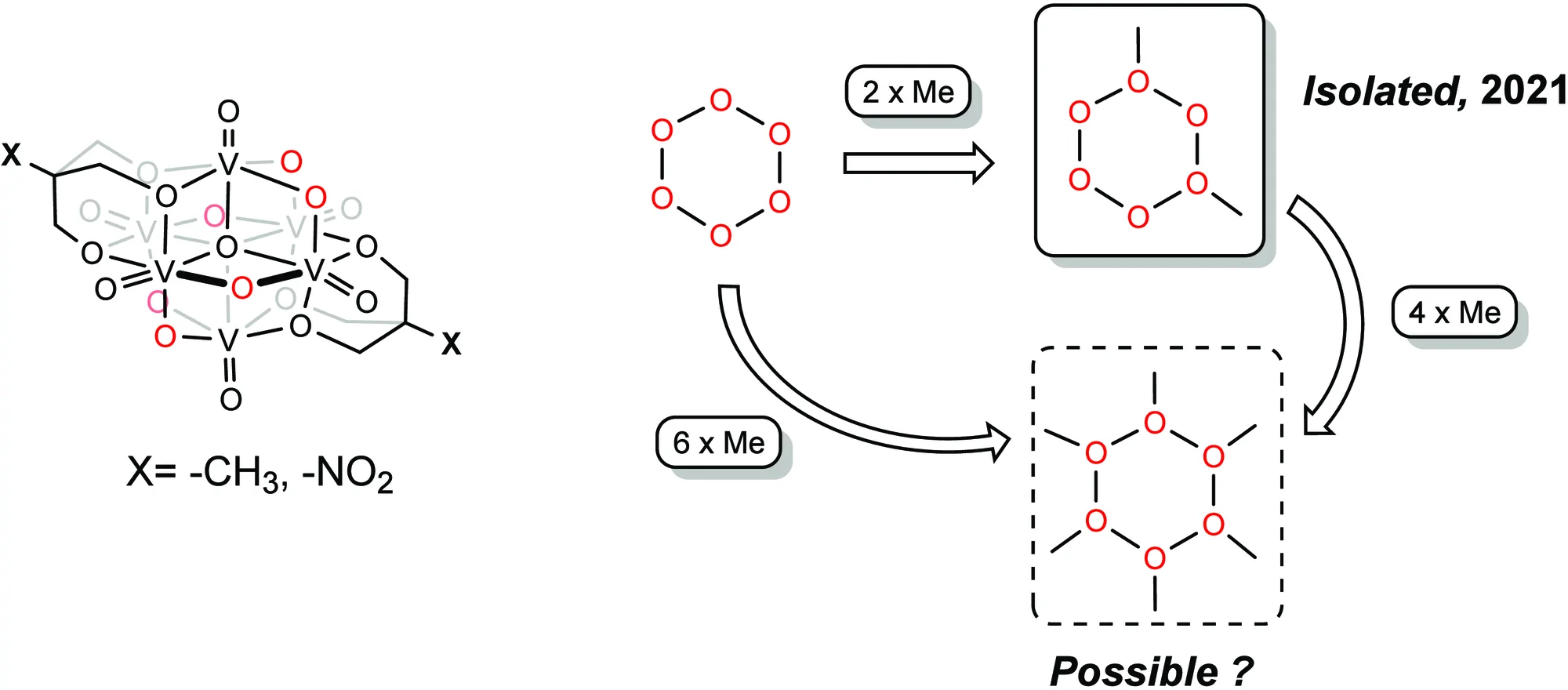
Structure of the [V^V^_6_O_13_(TRIOL^X^)_2_]^2–^ species (left) and the simplified representation of the six bridging μ_2_-oxo ligands applied to the methylation (right).

Besides modification of peripheral ligands, the oxide surface of Lindqvist-type polyoxovanadate clusters itself presents opportunities for chemical transformation. In particular, the bridging μ__2__-oxo ligands are known to be more basic than terminal vanadyl groups, rendering them nucleophilic sites that can participate in postsynthetic functionalization reactions.^26^ Alkylation of these bridging oxygen atoms provides access to a synthetic route that traverses the chemical space between polyoxovanadates and polyoxovanadate-alkoxide species (Figure 1, right).^20^

In the case of [V^V^_6_O_13_(TRIOL^X^)_2_]^2–^, six bridging μ_2_-oxo ligands are available for functionalization. However, experimental studies to date have been limited largely to partially alkylated derivatives, such as [V^V^_6_O_11_(OMe)_2_(TRIOL^X^)_2_]^2–^ and [V^V^_6_O_12_(OMe)­(TRIOL^X^)_2_]^−^, which have been experimentally isolated.^20^ These observations suggest that sequential methylation does not proceed through a simple stepwise process but instead occurs within a highly interconnected redox–alkylation network in which experimentally observed products represent only a subset of kinetically accessible intermediates. Many thermodynamically viable species are likely bypassed because of kinetic constraints or rapid downstream transformations, making it difficult to predict how redox state governs both the extent and regioselectivity of functionalization. This challenge motivates the present theoretical investigation.

Beyond structural modification, alkylation also provides a route to tune surface reactivity, particularly hydrogen-bonding ability and proton-coupled electron transfer (PCET) behavior. In polyoxovanadate clusters, the bridging μ_2_-O moieties are stronger hydrogen atom acceptors than the terminal vanadium-oxo groups. Therefore, the full alkylation of the [V_6_O_13_(TRIOL^X^)_2_]^2–^ species would direct the PCET reactivity toward the terminal vanadium-oxo groups similar to the one observed for polyoxovanadate-alkoxide clusters.^26–28^ Despite this appealing prospect, the extent to which successive alkylation steps can be achieved in a controlled manner remains unclear (Figure 1). This is primarily due to the interplay between cluster charge, redox state, and reaction energetics: as alkylation proceeds, the cluster becomes progressively less electron rich, raising the energetic cost of further functionalization and often requiring coupled alkylation and reduction events.^20^ This leads to a chemically rich landscape spanning species with varying numbers of methylated oxygen atoms and reduced vanadium centers, from the fully oxidized [V^V^_6_O_13_(TRIOL^X^)_2_]^2–^ to heavily reduced and alkylated derivatives such as [V^IV^_6_O_7_(OMe)_6_(TRIOL^X^)_2_]^2–^ species.

Here, we systematically explore this complex chemical space for a series of polyoxovanadate-alkoxide species. Density functional theory combined with microkinetic modeling is used to assess the thermodynamic and kinetic accessibility of [V^V^_6–*n*_V^IV^_*n*_O_13–*m*_(OCH_3_)_*m*_(TRIOL^X^)_2_]^*m*−*n*−2^ species with X = CH_3_ and X = NO_2_ formed via surface methylation and reduction reactions. To facilitate broader application of these insights, we further develop a simplified predictive framework based on Hammett parameters that capture key electronic trends without the need for explicit quantum mechanical calculations. This approach provides a practical tool for guiding future experimental and theoretical studies of functionalized polyoxovanadate clusters.

## Computational Details

All species were optimized without constraints using density functional theory as implemented in the ORCA 6.0.1 package.^29,30^ The calculations were carried out with the hybrid PBE0 exchange-correlation functional, the def2-TZVP basis set for vanadium and sulfur atoms, and the def2-SVP basis set for all other atoms.^31,32^ Dispersion effects were accounted for using the D4 Grimme correction.^33^ Solvent effects were accounted for using the implicit SMD solvation model with tetrahydrofuran parameters (THF).^34^ The nature of all stationary points and transition states was verified by the calculation of analytical vibrational frequencies. Partition functions were used in the computation of 298 K thermal contributions to free energy employing the usual ideal-gas, rigid-rotator, harmonic oscillator approximation.^35^ All reported reduction potentials are referenced against the ferrocene/ferrocenium (Fc^+^/Fc) couple in acetonitrile, which is −4.8 V.^36^ The impact of the basis set on the results is discussed in the Supporting Information (Section S1).

The microkinetic modeling simulations were performed using the Kinescope package.^37,38^ To obtain more reliable energetics, we performed single-point energy calculations using the def2-TZVP basis set for all atoms and the previously calculated thermodynamic corrections. The electron transfer steps depend on the strength of the reducing agent. To prevent reversible electron transfer steps and simplify the kinetic simulation, we assumed that a strong reducing agent was employed, rendering all reduction steps irreversible. While we acknowledge that the kinetics of electron transfer may vary across different POV species, such steps are generally faster than chemical transformations involving nuclear rearrangements.^39^ Therefore, the activation free energy for electron transfer processes was set at the diffusion limit, Δ*G* ^‡^_ET_ = 10 kcal/mol.

## Results and Discussion

### Polyoxovanadate’s Methylation and Reduction Chemical Space

The methylation process using methyl trifluoromethanesulfonate (CF_3_SO_3_CH_3_, also known as MeOTf) is a key strategy for the direct functionalization of the [V^V^_6_O_13_(TRIOL^X^)_2_]^2–^ species’ surface. Each methylation step increases the overall charge of the cluster, whereas each reduction step decreases the overall charge. Consequently, reaction pathways that alternate between methylation and reduction are generally required to maintain energetically accessible transformations. Therefore, this species could undergo up to six methylation reactions and up to six reduction events, leading to a vast chemical space. A clear and manageable nomenclature is key to identifying the different species in this space; thus, we label each species as *n*–*m*, where *n* indicates the number of reduction steps and *m* the number of methylation events both relative to the initial [V^V^_6_O_13_(TRIOL^X^)_2_]^2–^ species (Figure 2).

**Figure 2:**
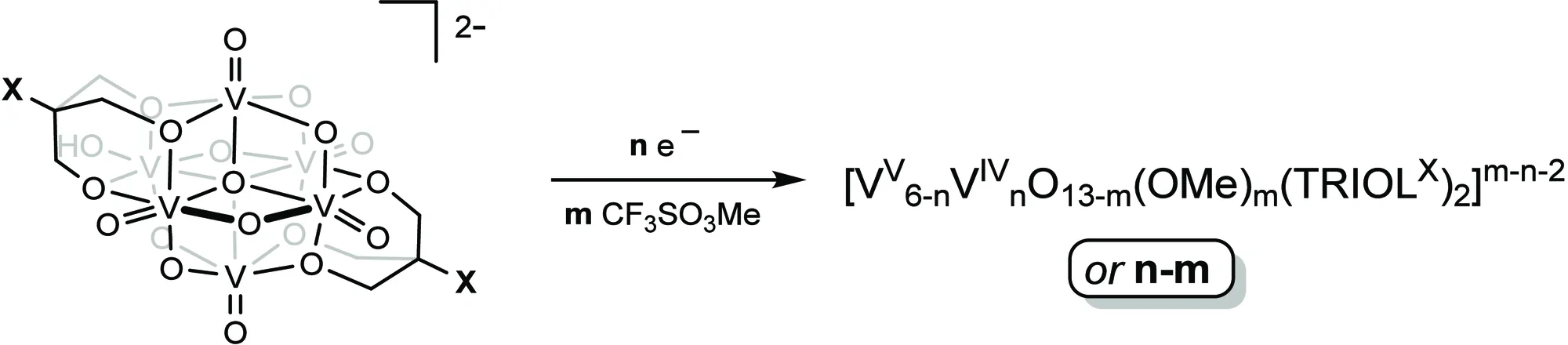
Structure of the [V^V^_6_O_13_(TRIOL^X^)_2_]^2–^ species with X = CH_3_ and NO_2_ (right) and the notation used to track reduction (*n*) and methylation (*m*) events.

Comparison between different TRIOL^X^ systems reveals clear substituent-dependent trends.^20^ Clusters bearing the methyl-substituted ligand preferentially undergo methylation, whereas those functionalized with the nitro-substituted ligand are more readily reduced. These trends are consistent with the electron-donating and electron-withdrawing character of the methyl and nitro TRIOL substituents, respectively.^25^

The initial speciation through two methylation steps and a single reduction event of the [V^V^_6_O_13_(TRIOL^Me^)_2_]^2–^ **(0–0)** species is presented in Figure 3. Two general trends are immediately apparent. First, reduction increases the nucleophilicity of the μ_2_-oxo ligands, thereby facilitating subsequent methylation. For example, methylation of the singly reduced species [V^V^_5_V^IV^O_13_(TRIOL^Me^)_2_]^3–^ (**1–0)** proceeds with a significantly lower activation barrier than methylation of the neutral species **0–0** (25.2 cf. 20.2 kcal/mol). This lower kinetic barrier is consistent with both our previous computational study and experimental observations.^20^ Second, successive methylation increases the overall positive charge of the cluster, making the resulting species more susceptible to reduction. This effect is reflected in the shift of the reduction potential from −1.0 V for **0–0** to −0.34 V for the singly methylated [V^V^_6_O_12_(OMe)­(TRIOL^Me^)_2_]^−^ (**0–1)** species which is also consistent with experimental observations.^20^

**Figure 3:**
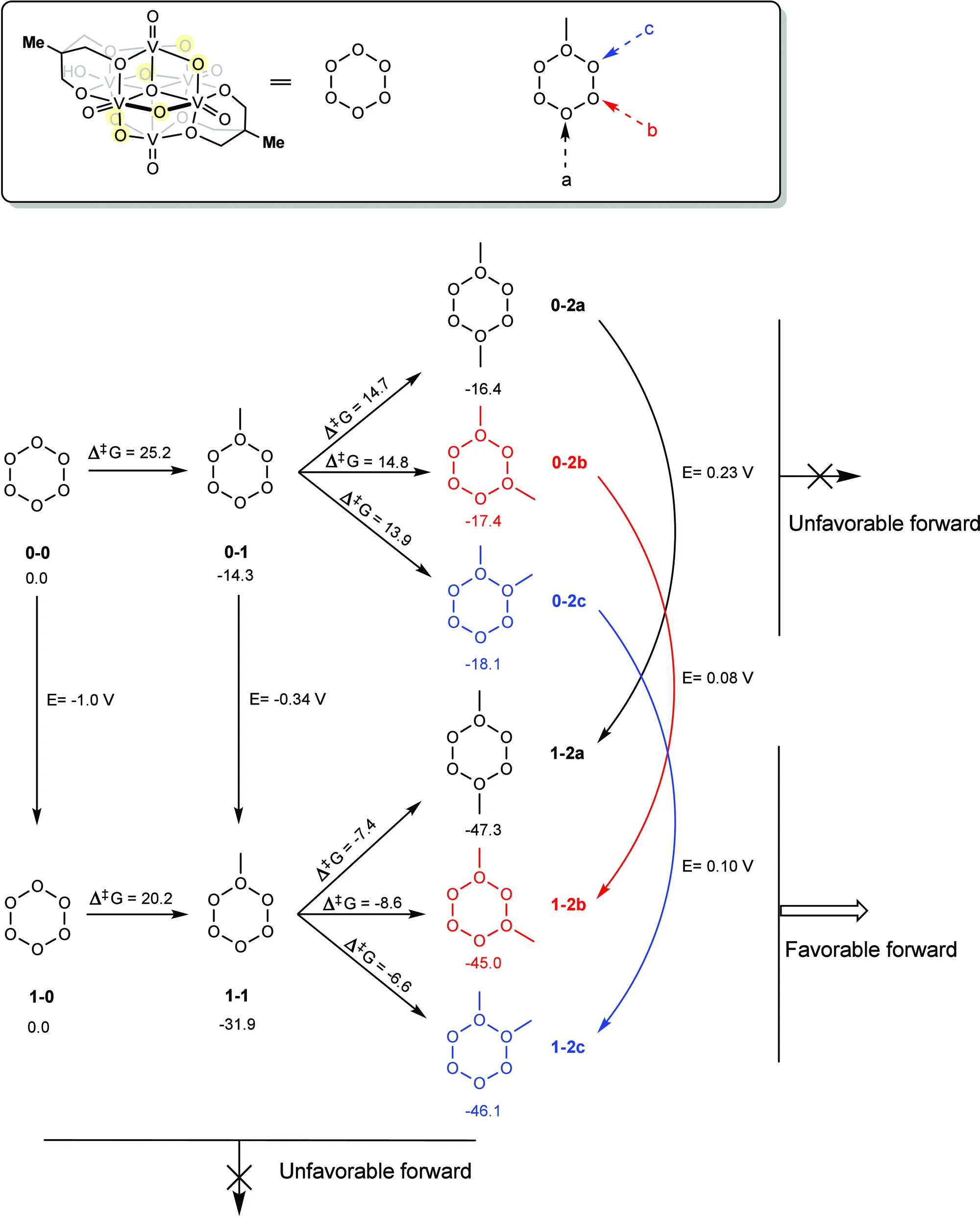
Initial electrochemical landscape of [V^V^_6_O_13_(TRIOL^Me^)_2_]^2–^ **(0–0)** species up to two methylations and one reduction. Reaction and activation Gibbs free energies in kcal/mol and redox potentials in Volts respect to Fc^+^/Fc.

Upon a second methylation event, three isomeric products (a, b, and c) become accessible with comparable thermodynamic stabilities and kinetic barriers. Reduction can proceed from any of these isomers, leading to intersecting pathways in which methylation and reduction sequences converge. Even at this limited level of functionalization, the chemical space already exhibits significant branching. To evaluate whether complete alkylation of the [V_6_O_13_(TRIOL^Me^)_2_]^2–^ cluster is feasible, we defined some criteria for identifying the experimentally accessible chemical space, namely, reduction potentials *E* >−1.0 V and activation barriers Δ*G* ^‡^ <29 kcal/mol.^40^ Applying these to the pathways shown in Figure 3 reveals an additional selectivity constraint. Specifically, a third methylation step is not energetically accessible from any of the doubly methylated **0–2** isomers. Instead, any progression beyond the [V^V^_6_O_11_(OMe)_2_(TRIOL^Me^)_2_] (**0–2)** species requires a reduction step, such that further methylation becomes feasible only after formation of the corresponding reduced [V^V^_5_V^IV^O_11_(OMe)_2_(TRIOL^Me^)_2_]^−^ (**1–2)** species. This requirement highlights the central role of the polyoxovanadate redox state in governing the sequence and selectivity of alkylation.

This interpretation is also consistent with previous experimental observations reported by the Matson group, where the doubly methylated [V^V^_5_V^IV^O_11_(OMe)_2_(TRIOL^Me^)_2_] (**0–2)** species was obtained under excess MeOTf without requiring prior reduction during the alkylation step.^20^ In contrast, our calculations indicate that further methylation beyond the **0–2** manifold becomes kinetically inaccessible in the absence of reduction, as all pathways leading to the third methylation exceed the adopted activation barrier threshold. These species therefore fall within the kinetic shadow region (discussed later on the Microkinetic Modeling section), where thermodynamically accessible intermediates fail to accumulate under experimentally relevant conditions. For the NO_2_-substituted system, the second methylation barrier is calculated to be slightly higher (ΔΔ^‡^ *G* = 30 kcal/mol), suggesting a greater dependence on prior reduction to enable alkylation. It should be noted that these calculated free-energy barriers correspond to standard-state conditions (1.0 M, 298 K); therefore, variations in reagent concentration or temperature may substantially influence the experimentally observed reactivity.

Building on these results, we systematically explored all possible combinations of methylation and reduction steps to map the full energetic landscape of this chemical space. At the second, third, and fourth methylation levels, each species can exist as three distinct isomers, each of which can undergo further methylation or reduction through nonequivalent pathways. Consequently, a scheme such as the one presented in Figure 3 for the complete chemical space becomes extremely complex. Therefore, the complete speciation pathways of [V_6_O_13_(TRIOL^Me^)_2_]^2–^ and [V_6_O_13_(TRIOL^NO2^)_2_]^2–^ species have been included in the Supporting Information (Figures S1–S4 and Tables S1 and S2). When the previously mentioned thresholds limit the speciation of experimentally accessible species, multiple viable pathways remain beyond doubly methylated species with different extents of methylation depending on the reduction-methylation reaction sequence and overall conditions (Figure S5). These results support the experimental feasibility of achieving full methylation, while also indicating that partially methylated intermediates can be selectively accessed through appropriate control of reaction conditions, specifically the reducing strength and amount of reducing agent as well as the reaction order between the reducing and methylating agents. Since the reduction events require a specific redox reagent, the choice of reducing agent can be strategically tuned to selectively enable desired reduction steps while suppressing others. The distribution of reduction potentials of the species with methylated and nitrated TRIOLs show many species with experimentally accessible reduction events (Figure S6). Notably, the presence of the nitro group consistently shifts the reduction potential by approximately +0.42 V compared to the methyl analogue (Figure S7). This shift indicates a consistent electronic effect imparted by the electron-withdrawing NO_2_ group, which facilitates the cluster reduction. It also indicates that the Hammett parameter could be used as a descriptor of the effect of TRIOL substituent into the cluster properties.

### Regioselectivity Control with Reduction States

After mapping the full reaction landscape for successive methylation, we next examined whether the regioselectivity of the second methylation step depends on the redox state of the monomethylated intermediate **1–0**. The three possible doubly methylated products differ only in the identity of the μ_2_-oxo group undergoing alkylation, and their relative formation is therefore determined by the local electronic structure of the oxo sites. Figure 4 presents the dominant doubly methylated isomer at different redox states, as determined from the calculated activation barriers for formation of each isomer (see the first column of Table S1 in the Supporting Information (SI) for details). In the absence of a reduction event, as well as for the doubly and triply reduced species, the methylation at the μ_2_-oxo site leading to isomer c proceeds with the lowest activation barrier, indicating that this site remains the most reactive under these conditions. In contrast, one and five electron reduced species shift the kinetic preference toward the formation of isomer b, consistent with a redistribution of electron density across the bridging oxo framework. Upon four-electron reduction, methylation at the site corresponding to isomer a becomes kinetically favored.

**Figure 4:**
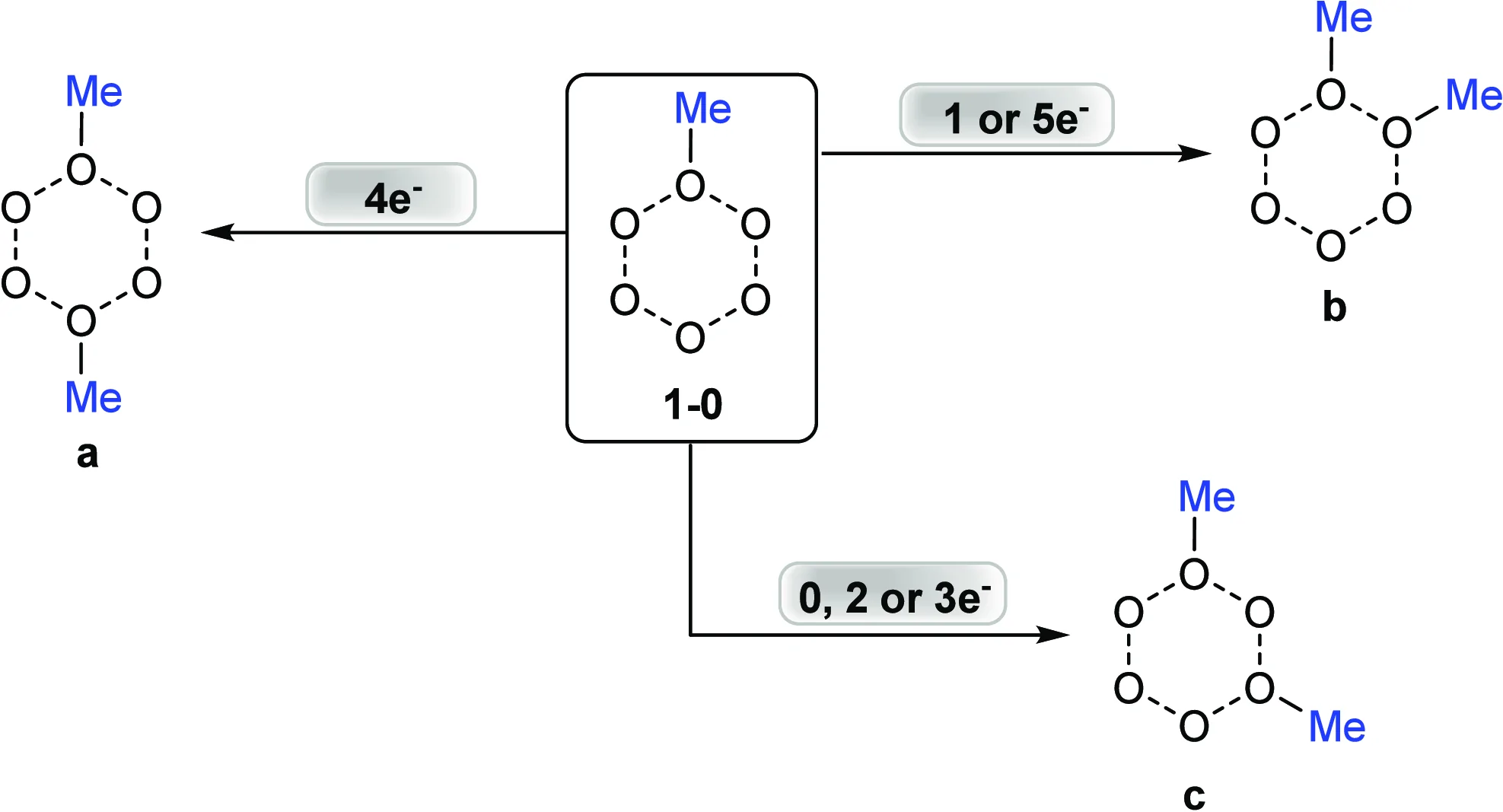
Reduction-state dependence of methylation selectivity for TRIOL^Me^ cluster.

Notably, for the TRIOL^Me^ system, the calculated preference for formation of isomer c under the neutral and doubly reduced states is consistent with the available experimental observations, where the corresponding demethylated product was identified as the predominant species. This agreement suggests that the regioselectivity is primarily governed by the intrinsic electronic structure of the bridging μ_2_-oxo sites and can be reasonably captured by the calculated reaction barriers.

Additional support for this interpretation is provided by the calculated spin density distributions of the reduced nonmethylated clusters (Figure S21 in the Supporting Information). The added electron density localizes preferentially on specific vanadium centers in the computed electronic ground state, electronically differentiating nearby bridging μ_2_-oxo ligands. However, this localization should not be interpreted as completely static, as redistribution of electron density may occur in response to structural perturbations such as methylation or changes in coordination environment.^41^ Nevertheless, the calculated spin density distributions support the conclusion that reduction does not uniformly activate all μ_2_-oxo groups, but instead selectively enhances the nucleophilicity of specific sites depending on the overall electron count. Consequently, the regioselectivity of methylation can be altered by adjusting the reduction state of the cluster prior to alkylation.

### Microkinetic Modeling

The complete reaction network connecting the initial [V^V^_6_O_13_(TRIOL^X^)_2_]^2–^ to fully methylated species spans an extensive chemical space, comprising more than 100 distinct methylation and reduction transformations with frequent intersections between the two processes. While this global network provides a comprehensive energetic picture, it is not well suited for identifying conditions that enable selective partial methylation. Previous experimental studies have shown that such selectivity is most effectively achieved through stepwise protocols in which reduction and methylation are separated into a multistep process.

To directly probe the kinetic origins of selectivity, we therefore focus on a restricted but chemically representative subset of the chemical space. Specifically, we examine a subspace comprising three successive methylation levels combined with up to two reduction events, starting from the parent cluster **0–0** (Figure 5). This subspace captures the early-stage transformations most relevant to experimental functionalization, while remaining tractable for microkinetic modeling.

**Figure 5:**
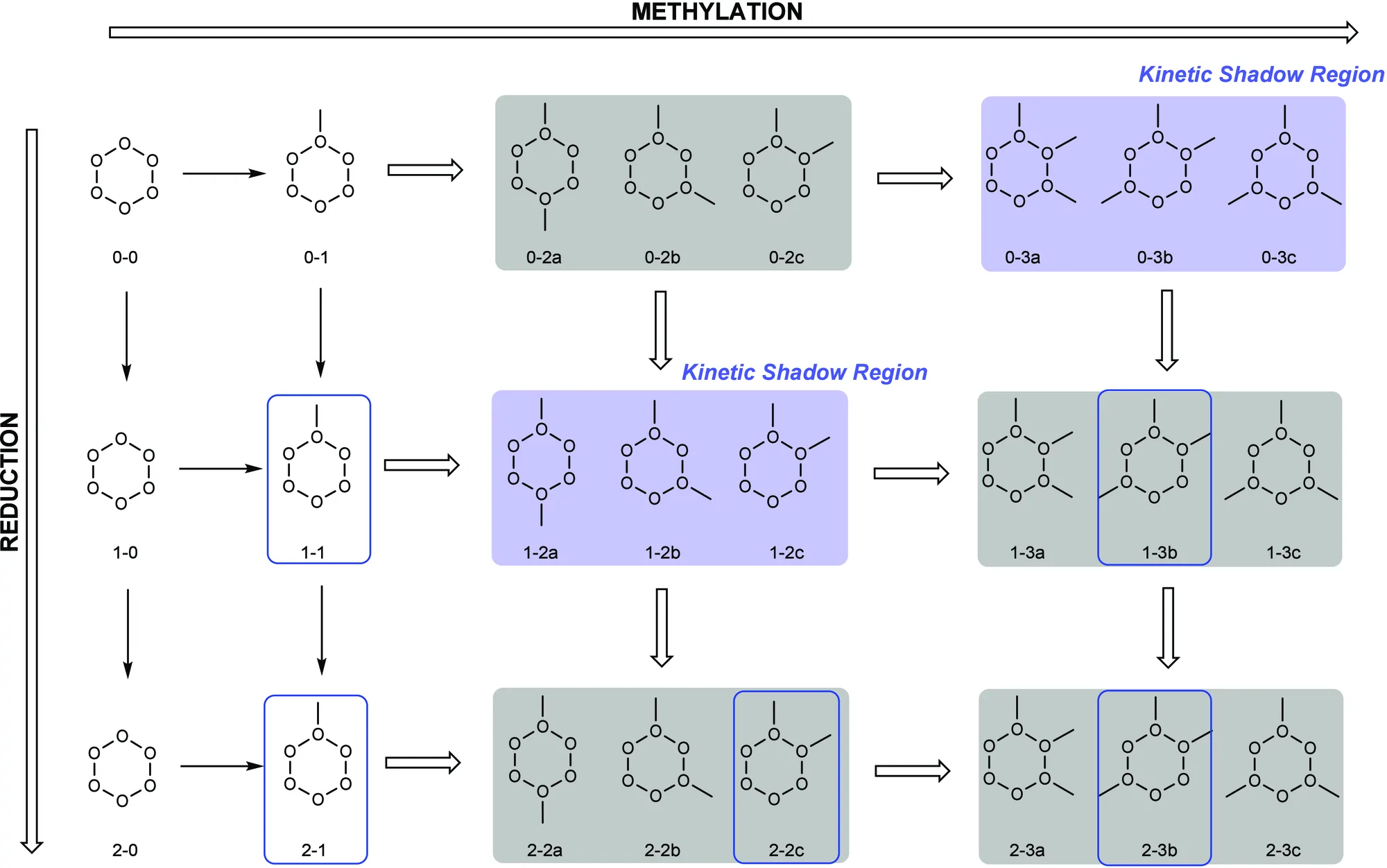
Speciation subspace up to three successive methylation events and up to two reduction events starting at cluster **0–0**. The kinetic shadow region (purple) denotes areas of the reaction network where intermediates are not kinetically accessible or do not build up to observable concentrations. Blue circles indicate favorable synthetic targets.

As expected, increasing the availability of MeOTf biases the network toward higher methylation states, whereas increasing the electron input promotes reduction. This behavior is most transparent for the first methylation step. The methylation of **0–0** proceeds with a moderate barrier (Δ*G* ^‡^ = 24.8 kcal/mol), and this barrier decreases substantially upon reduction, consistent with enhanced μ_2_-oxo nucleophilicity. Consequently, selective access to different monomethylated species is readily achieved by tuning the reduction level prior to alkylation. For example, microkinetic simulations show that **0–1** species dominate under purely methylating conditions with a ratio of reducing agent, MeOTf, and [V^V^_6_O_13_(TRIOL^X^)_2_]^2–^ of 0:1:1, **1–1** species become predominant at a 1:1:1 ratio, and [V^V^_4_V^IV^_2_O_12_(OMe)­(TRIOL^Me^)_2_]^3–^ (**2–1)** species emerges at higher electron loadings in a 2:1:1 ratio (Figures S11–S13).

However, the kinetic behavior changes markedly upon introduction of the second methylation step. In several regions of the grid, doubly methylated intermediates are consumed faster than they accumulate, preventing their experimental observation. Notably, all three isomers of the **1–2** cluster are predicted to be short-lived across a wide range of reagent ratios. Even under conditions that might intuitively favor partial methylation, the simulations indicate rapid progression to further methylation, with [V^V^_5_V^IV^O_10_(OMe)_3_(TRIOL^Me^)_2_] (**1–3b**) species emerging as the dominant product (Figure S14).

This behavior highlights an important kinetic constraint: When reduction precedes or accompanies the second methylation, the increased reactivity of the cluster drives immediate overfunctionalization. As a result, selective isolation of doubly methylated species requires a stepwise synthetic sequence, in which two methylation events are first carried out on the unreduced cluster (**0–0** → **0–2**), followed by reduction. This strategy avoids the kinetic acceleration that otherwise funnels the system toward higher methylation states and is consistent with experimental protocols used to isolate partially alkylated POM clusters. Furthermore, within the studied subspace, we identify regions where intermediates fail to accumulate due to rapid downstream conversion. We refer to these regions as kinetic shadow zones (purple circles in Figure 5). In contrast, other regions are bounded by kinetic bottlenecks, where large activation barriers or unfavorable redox potentials effectively halt further reactivity. For instance, direct methylation of **0–2** to form **0–3** isomers requires activation barriers exceeding 30 kcal/mol, rendering these pathways inaccessible in the absence of prior reduction.

Conversely, several regions of the subspace correspond to conditions where specific species dominate the simulated product distributions (blue circles in Figure 5). Although the present model does not incorporate solubility or crystallization effects, this framework demonstrates how combining computed energetics with microkinetic modeling can identify viable synthetic targets, rationalize experimentally observed selectivity, and delineate regions of the reaction network where selective access is intrinsically unlikely.

### Hammett-Guided Prediction of Electronic Effects in Polyoxovanadates

The speciation chemical space of [V^V^_6_O_13_(TRIOL^X^)_2_]^2–^ species contain 91 minima and 147 transition states for each substituent, raising how to leverage this data to study similar systems since experimental studies often explore modified structures to tune reactivity.^42^ This process introduces a substantial burden, as it requires monitoring and tracking hundreds of calculations, which can become cumbersome and tedious, thereby limiting practical utility. Recently, Batista and co-workers^43^ successfully trained neural networks to predict photoredox potentials using Hammett parameters, achieving high accuracy. In a similar vein, our redox data has a linear correlation with Hammett parameters, analogous to those commonly observed in standard organic molecules for reduction potentials (Figure S7 in the SI). Building on this observation, we propose a method that leverages the linear relationship between thermodynamic and kinetic parameters, obtained from DFT, and Hammett constants to estimate the electronic properties of analogous substituents. This approach offers a promising pathway to obtain reliable, DFT-like predictive values without the need for exhaustive quantum chemical calculations or machine learning model training.

It is important to notice that while there is a strong linear correlation between the redox potentials and the Hammet parameters, deviations begin to emerge at more extreme reduction potentials, particularly when *E* <−3.0 V vs Fc^+^/Fc. This suggests a breakdown of the correlation in that regime, likely due to a shift in the underlying redox processes. For example, the reduction of neutral species is not expected to mirror the behavior of a highly anionic species. This divergence underscores the limitations of simple substituent-based correlations under extreme electronic conditions. As a result, to ensure the highest predictive accuracy, each step of the computational process must be evaluated individually, with careful attention to maximizing structural and electronic analogy when extending the scope of substitution beyond the symmetrical TRIOL methyl and nitro cases. As a starting point, we assess the reliability of predicting the reduction potential for reducing the **0–0** cluster. Using computed data from two symmetrical TRIOL^X^ clusters (with X = −CH_3_ and −NO_2_), we construct a linear correlation between the reduction potential and the total Hammett parameter (σ),^44^ defined as the sum of the individual σ-values for each substituent within the TRIOL group.

Figure 6 demonstrates that the explicitly computed reduction potentials of the reference cluster **0–0** are highly correlated with Hammett σ constants (*R* ^2^ = 0.99). We leveraged this relationship to efficiently predict the potentials of the **3–0** cluster series. Using only two calculated anchor points (X,Y = Me and X,Y = NO_2_), an extrapolated regression line (Figure 6, orange line) was generated, allowing the reduction potentials of the remaining **3–0** derivatives to be determined solely from their Hammett parameters. As detailed in Table 1, these extrapolated values are in excellent agreement with full SMD­(THF)-PBE0-D4/BS1 calculations, exhibiting deviations of less than 0.1 V.

**Figure 6:**
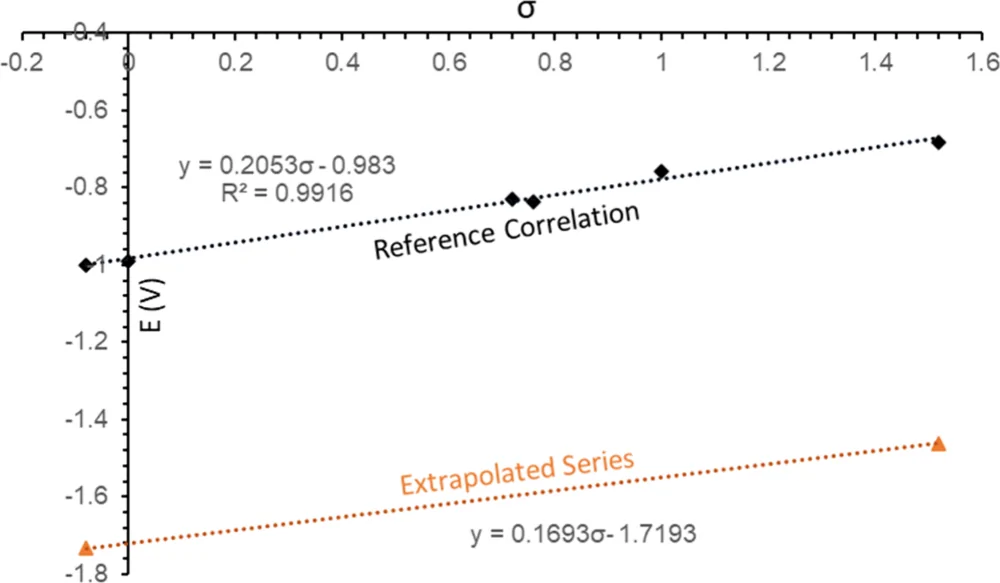
Correlation between Hammett parameters and the reduction potentials of complex **0–0** and its derivatives (black line) and complex **3–0** and its derivatives for extrapolation (orange line), calculated at the SMD­(THF)-PBE0-D4/BS1 level of theory.

**Table 1:** Comparison of the Reduction Potentials (V vs Fc^+^/Fc) for Cluster **3–0** and Its Derivatives, Obtained via Hammett Plot Extrapolation and Calculated at the SMD­(THF)-PBE0-D4/BS1 Level of Theory

substituents (X, Y)	σ	predicted *E* (V)	computed *E* (V)
X = Br, Y = Br	1.00	–1.55	–1.53
X = H, Y = H	0	–1.72	–1.74
X = Me, Y = NO_2_	0.72	–1.60	–1.60
X = H, Y = NO_2_	0.76	–1.60	–1.68
X = Cl, Y = NO_2_	1.23	–1.51	–1.49
X = Cl, Y = Cl	0.94	–1.56	–1.51

Since the linear correlation was constructed using data calculated using the def2-TZVP basis set for vanadium and sulfur and def2-SVP for all the other elements, the predicted values inherently reflect the accuracy of that basis set. However, these predictions can be translated to a higher-level basis set via an additional linear regression between data at different basis set levels, as discussed in the Supporting Information Section S1.

We next examine whether this Hammett-based predictive model can reproduce broader trends in methylation thermodynamics and kinetics, beyond the methyl and nitro substituents. The computed data for a variety of substituents show a strong linear relationship for methylation thermodynamics, with *R* ^2^ = 0.98 (Table S3 and Figure S15). In contrast, the correlation for methylation kinetics is slightly weaker, with *R* ^2^ = 0.93 (Table S4 and Figure S16). However, the activation barriers across the substituent series differ by less than ∼1 kcal/mol in many cases, making the correlation more sensitive to the intrinsic uncertainty of the DFT calculations. Thus, the lower *R* ^2^ value does not indicate a breakdown of the Hammett relationship but rather reflects the difficulty of resolving very small energetic differences. Consistent with this interpretation, applying triple-ζ single-point energy corrections improves the kinetic correlation to *R* ^2^ = 0.98 (Figure S17), indicating that the higher-level basis set better captures these subtle energetic variations.

Overall, these examples demonstrate that Hammett parameters can be reliably used to predict computed values with high accuracy. Although the data set examined here includes only a small number of representative substituents, the results highlight how accurate and convenient this approach can be as a computational shortcut for estimating high-quality energetic data.

## Conclusions

We presented a systematic computational study of the complete chemical space of [V^V^_6–*n*_V^IV^_*n*_O_13–*m*_(OCH_3_)_*m*_(TRIOL^X^)_2_]^*m*−*n*−2^ species, focusing on methyl and nitro-substituted TRIOLs. The resulting energetic profiles, combined with microkinetic modeling, provide effective guidance for experimental efforts aimed at selectively controlling the degree of methylation and reduction. This approach reveals regions of kinetic inaccessibility, so-called kinetic shadow region, which may hinder product formation and should therefore be avoided during synthetic design. Given the large computational cost associated with expanding our study to the chemical spaces of derived species, we introduce a Hammett parameter-based model that enables rapid estimation of redox and methylation behavior. This strategy offers a practical shortcut for predicting key energetic trends with high reliability, and we hope it will prove useful in guiding future experimental and computational explorations of polyoxovanadate clusters and related systems.

## Supplementary Material



## Notes

The optimized coordinates, all inputs and outputs for all calculations can be found in FigShare (https://doi.org/10.6084/m9.figshare.30182710). Complete speciation chemical space for the methylation and reduction of [V_6_O_13_(TRIOL^Me^)_2_]^2–^ and [V_6_O_13_(TRIOL^NO2^)_2_]^2–^ species, Hammet correlation plots, microkinetic modeling results, and basis set effects (PDF)
