# Supplementary material for: Redox-State-Driven Site Selectivity and Kinetic Gating in Polyoxovanadate Methylation
Source: Inorg Chem. 2026 Aug 6;65(32):18563–71. doi: 10.1021/acs.inorgchem.6c01638 (PMC13488394; doi:10.1021/acs.inorgchem.6c01638)
Supplement: Supplementary file 1 [file ic6c01638_si_001.pdf]

Supporting Information

# Redox-State Driven Site Selectivity and Kinetic Gating in Polyoxovanadate Methylation

Nghia Le and Pere Miró*

Department of Chemistry, University of Iowa, Iowa City, Iowa 52240, United States

Email: pere-miro@uiowa.edu

fafa

All inputs and outputs for all calculations can be found in FigShare

https://doi.org/10.6084/m9.figshare.30182710

## Contents

**S.1 Basis Set Effect** ...................................................... **S32**

## Figure

**Figure S1.** Full methylation and reduction speciation chemical space starting at $[V_6O_{13}(TRIOL^{Me})_2]^{2-}$cluster, computed at PBE0-D4-SMD(ACN)/BS1 level theory. Gibbs free energies in kcal/mol and reduction potential in Volts respect the $Fc^+/Fc$ reference. ...................................................... S7

**Figure S2.** Reduction potentials for 0-2, 0-3, and 0-4 intermediates with $TRIOL^{Me}$ associated with their isomers, computed at PBE0-D4-SMD(ACN)/BS1 level theory. Reduction potential in Volts respect the $Fc^+/Fc$ reference. ...................................................... S8

**Figure S3.** Full methylation speciation chemical space starting at $[V_6O_{13}(TRIOLNO2)]^{2-}$cluster, computed at PBE0-D4-SMD(ACN)/BS1 level theory. Gibbs free energies in kcal/mol and reduction potential in Volts respect the $Fc^+/Fc$ reference. ...................................................... S12

**Figure S4.** Reduction potentials for 0-2, 0-3, and 0-4 intermediates with $TRIOLNO2$ associated with their isomers, computed at PBE0-D4-SMD(ACN)/BS1 level theory. Reduction potential in Volts respect the $Fc^+/Fc$ reference. ...................................................... S13

**Figure S5.** Full methylation and reduction speciation chemical space starting at $[V_6O_{13}(TRIOL^{Me})_2]^{2-}$cluster, highlighting the favorable steps that remain visible under conditions with reduction potentials E < –1.0 V vs $Fc^+/Fc$ and activation barriers $\Delta^{\ddagger}G$ > 29 kcal/mol. ...................................................... S17

**Figure S6.** Distribution of the reduction potentials of $TRIOL^X$-substituted clusters (X = $–CH_3$ and $–NO_2$) across studied chemical space. ...................................................... S18

**Figure S7.** Correlation between the redox potentials of $TRIOL^X$-substituted clusters with X = $–CH_3$ and $–NO_2$. ...................................................... S19

**Figure S8.** The correlation of reduction potential correlation calculated SMD(THF)-PBE0-D4/BS1 and SMD(THF)-PBE0-D4/def2-TZVP//BS1 level of theory. The data for this plot is tabulated in Table S7....... S20

**Figure S9.** The correlation of reduction potential correlation calculated SMD(THF)-PBE0-D4/BS1 and SMD(THF)-PBE0-D4/def2-TZVP//BS1 level of theory. The data for this plot is tabulated in Table S7....... S21

**Figure S10.** Energetic profile calculated at SMD(THF)-PBE0-D4/def2-TZVP//SMD(THF)-PBE0-D4/BS1 level theory. This scheme is used for the microkinetic modeling presented in Figure 4 ...................................................... S22

**Figure S11.** The microkinetic modelling results for the subspace presented in Figure 5. Ratio of reducing agent, MeOTf, and $[V^V{}_6O_{13}(TRIOL^X)_2]^{2-}$ was set to 0:1:1 ...................................................... S23

**Figure S12**. The microkinetic modelling results for the subspace presented in Figure 5. Ratio of reducing agent, MeOTf, and $[V^V{}_6O_{13}(TRIOL^X)_2]^{2-}$ was set to 1:1:1 ...................................................... S24

**Figure S13.** The microkinetic modelling results for the subspace presented in Figure 5. Ratio of reducing agent, MeOTf, and $[V^V{}_6O_{13}(TRIOL^X)_2]^{2-}$ was set to 2:1:1 ...................................................... S25

**Figure S14.** The microkinetic modelling results for the subspace presented in Figure 5. Ratio of reducing agent, MeOTf, and $[V^V{}_6O_{13}(TRIOL^X)_2]^{2-}$ was set to 1:2:1 ...................................................... S26

**Figure S15.** Correlation between Hammett parameters and thermodynamic methylation of complex 0-0 and its derivatives calculated at the SMD(THF)-PBE0-D4/BS1 level of theory. ...................................................... S28

**Figure S16**. Correlation between Hammett parameters and kinetics methylation of compound 0-0 and its derivatives calculated at the SMD(THF)-PBE0-D4/BS1 level of theory. ...................................................... S30

**Figure S17.** Correlation between Hammett parameters and kinetics methylation of compound 0-0 and its derivatives calculated at the the SMD(THF)-PBE0-D4/def2-TZVP//BS1 level of theory. ........................... S31
**Figure S18.** Correlation of the thermodynamic energies for the first methylation step in Table S5 between the SMD(THF)-PBE0-D4/BS1 and SMD(THF)-PBE0-D4/def2-TZVP//BS1 level of theory. ........................... S34
**Figure S19.** Correlation of the thermodynamic energies for the second methylation step in Table S5 between the SMD(THF)-PBE0-D4/BS1 and SMD(THF)-PBE0-D4/def2-TZVP//BS1 level of theory. ..................... S35
**Figure S20.** Correlation of the thermodynamic energies for the third methylation step in Table S5 between the SMD(THF)-PBE0-D4/BS1 and SMD(THF)-PBE0-D4/def2-TZVP//BS1 level of theory. ........................... S36
**Figure S21.** Spin Density Plots for Different Reduction States of the Nonmethylated $TRIOL^{Me}$ Cluster ..... S37

## Scheme

**Scheme S1.** Simplified methylated polyoxovanadate species associated with the compound label. ............... S6

## Table

**Table S1.**Transition state energetics of methylation $[V_6O_{13}(TRIOL^{Me})_2]^{2-}$cluster, computed at PBE0-D4-SMD(ACN)/BS1 level theory. Gibbs free energies in kcal/mol. ..................................................................... S9
**Table S2.**Transition state energetics of methylation $[V_6O_{13}(TRIOLNO2)]^{2-}$cluster, computed at PBE0-D4-SMD(ACN)/BS1 level theory. Gibbs free energies in kcal/mol. .................................................................. S14
**Table S3.** Computed thermodynamics for the first methylation step of various substituents, calculated at the SMD(THF)-PBE0-D4/BS1 level of theory. ........................................................................................ S27
**Table S4.** Computed kinetics for the first methylation step of various substituents, calculated at the SMD(THF)-PBE0-D4/BS1 level of theory. ........................................................................................ S29
**Table S5.** Thermodynamic energetics of the methylation of the $[V_6O_{13}(TRIOLMe)]^{2-}$ cluster, from compound **0-0** to compound **0-3**, computed at PBE0-D4-SMD(ACN)/BS1 level theory ............................................... S33
**Table S6.** Computed results for transition state energies of selected methylation processes used to examine the effect of basis set choice. ........................................................................................................... S38
**Table S7**. Computed results for selected reduction processes used to examine the effect of basis set choice. $\Delta G_1$–BS1 values were calculated at the SMD(THF)-PBE0-D4/BS1 level of theory, while $\Delta G_1$–TZ values were computed at the SMD(THF)-PBE0-D4/def2-TZVP//BS1 level of theory..................................................... S39
**Table S8.** Computed results for the methylation processes used in microkinetic modeling presented in Figure 4.. ......................................................................................................................................... S40

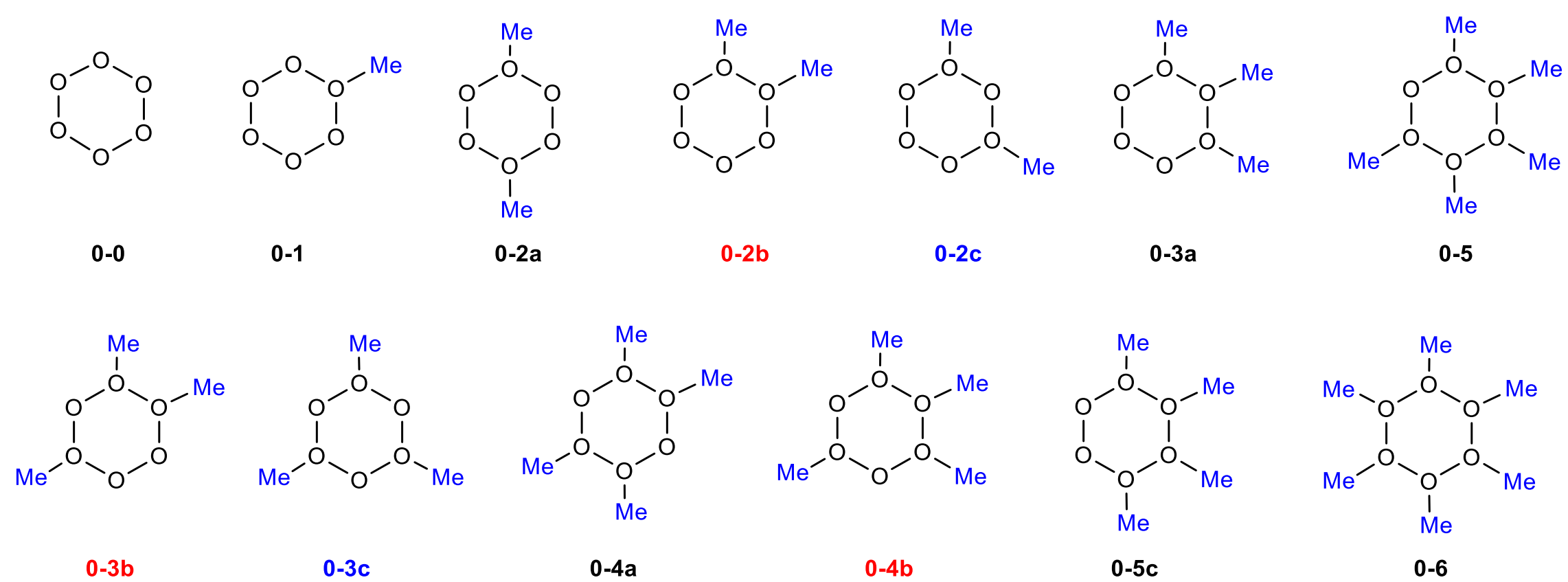


**Scheme S1.** Simplified methylated polyoxovanadate species associated with the compound label.

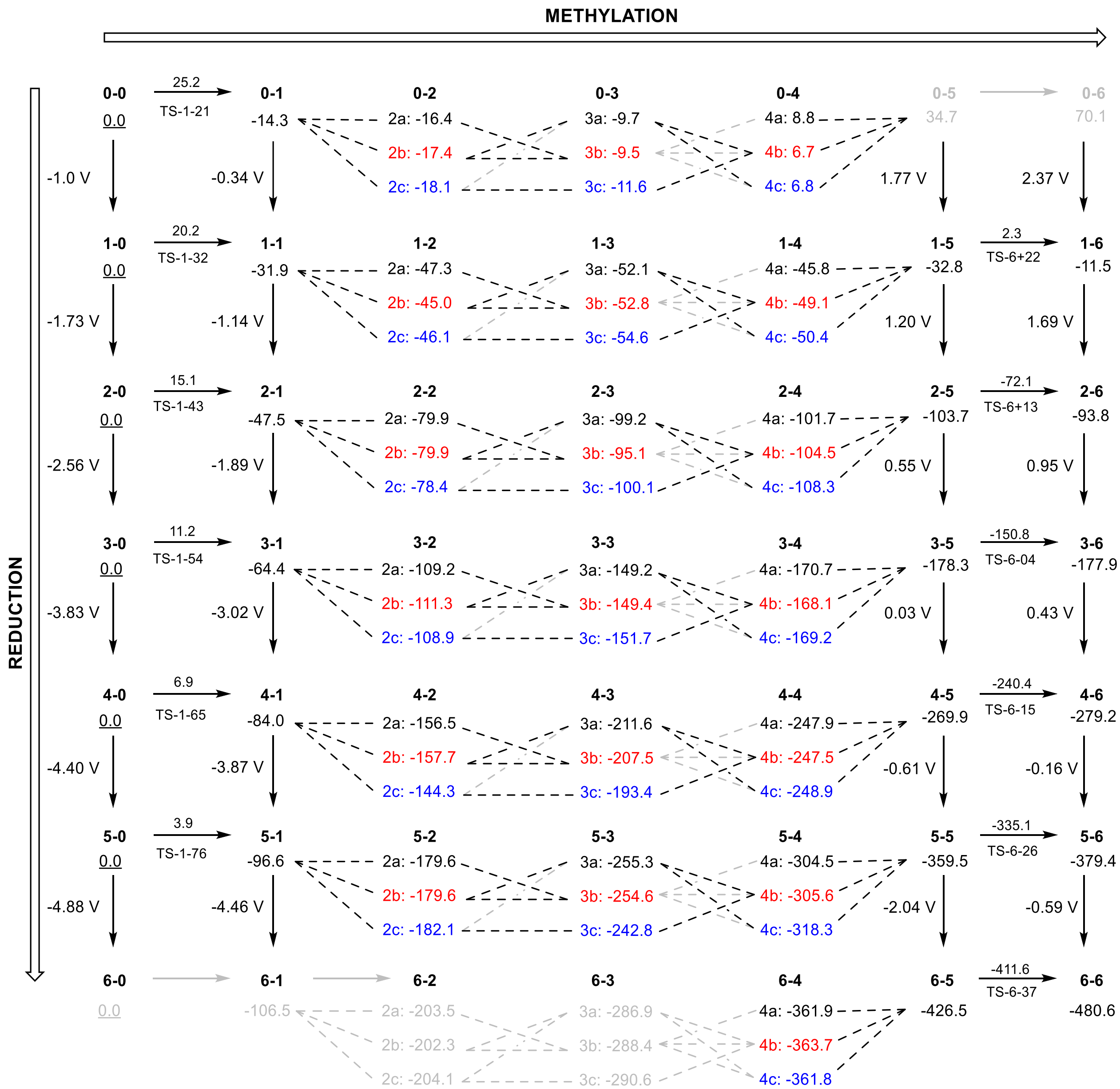


**Figure S1.** Full methylation and reduction speciation chemical space starting at $[V_6O_{13}(TRIOL^{Me})_2]^{2-}$cluster, computed at PBE0-D4-SMD(ACN)/BS1 level theory. Gibbs free energies in kcal/mol and reduction potential in Volts respect the $Fc^+/Fc$ reference.

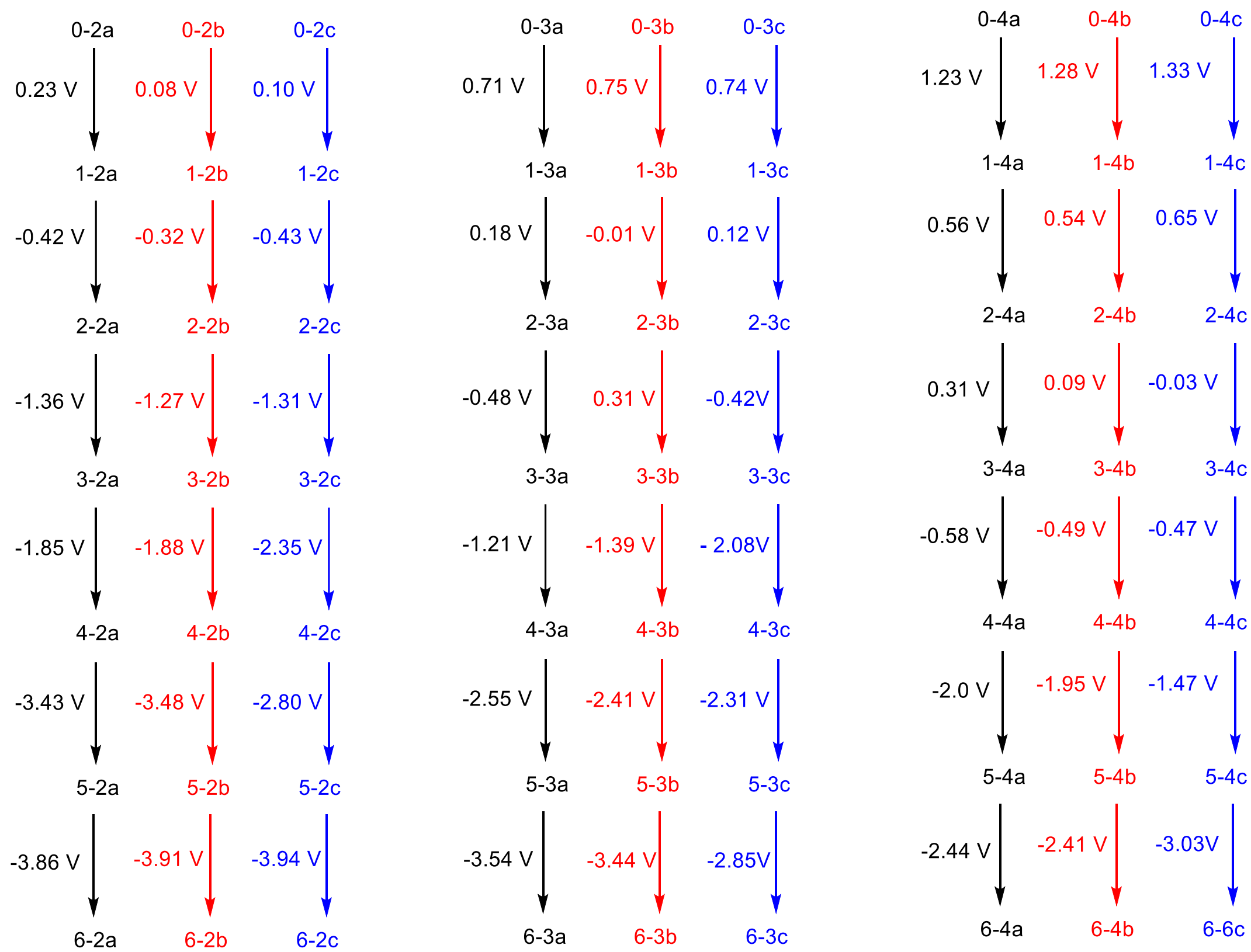


**Figure S2.** Reduction potentials for 0-2, 0-3, and 0-4 intermediates with TRIOL$^{Me}$ associated with their isomers, computed at PBE0-D4-SMD(ACN)/BS1 level theory. Reduction potential in Volts respect the $Fc^+$/Fc reference.

**Table S1.** Transition state energetics of methylation $[V_6O_{13}(TRIOL^{Me})_2]^{2-}$ cluster, computed at PBE0-D4-SMD(ACN)/BS1 level theory. Gibbs free energies in kcal/mol.

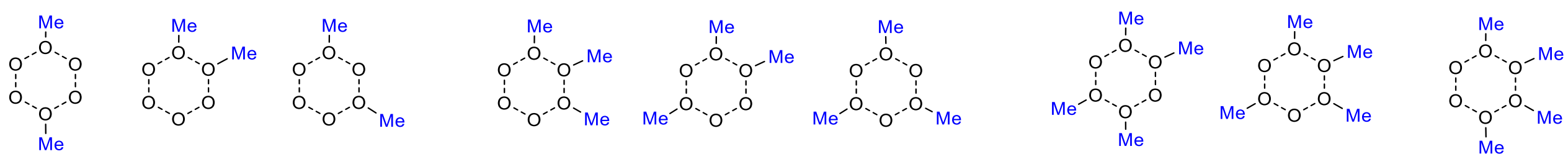

2a-01 2b-01 2c-01 3a+11 3b+11 3c+11 4a+21 4b+21 4c+21

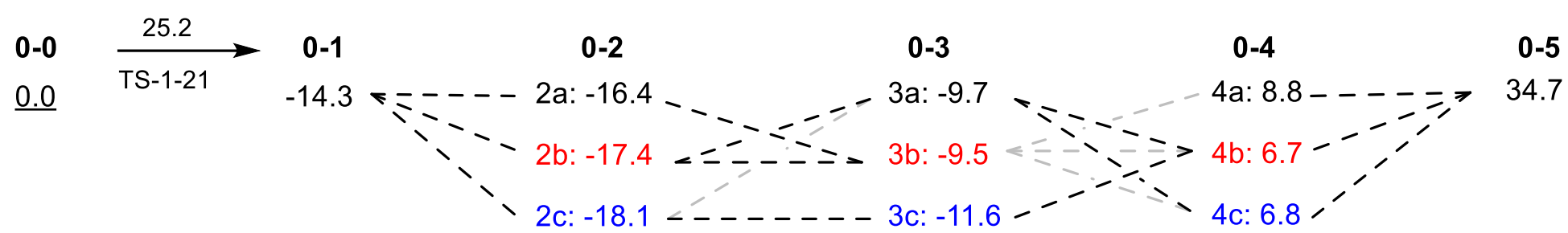


| **0-1** to **0-2** | **0-2** to **0-3** | **0-3** to **0-4** | **0-4** to **0-5** |
|---|---|---|---|
| TS-2a-11: 14.7 | TS-2a-3b-01: 14.7 | TS-3a-4b+11: 26.4 | TS-5a-21: 48.2 |
| TS-2b-11: 14.8 | TS-2b-3a-01: 13.3 | TS-3a-4c+11: 26.1 | TS-5b-21: 47.3 |
| TS-2c-11: 13.9 | TS-2b-3b-01: 15.1 | TS-3b-4a+11: 28.0 | TS-5c-21: 48.3 |
| | TS-2c-3a-01: 13.5 | TS-3b-4b+11: 27.0 | |
| | TS-2c-3b-01: 15.4 | TS-3b-4c+11: 26.2 | |
| | TS-2c-3c-01: 13.8 | TS-3c-4b+11: 25.0 | |

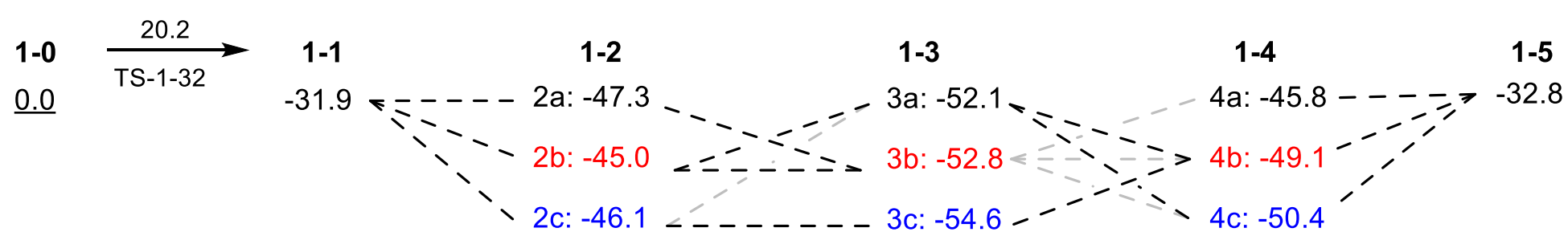


| **1-1** to **1-2** | **1-2** to **1-3** | **1-3** to **1-4** | **1-4** to **1-5** |
|---|---|---|---|
| TS-2a-22: -7.4 | TS-2a-3b-12: -20.1 | TS-3a-4b-02: -25.2 | TS-5a-12: -13.5 |
| TS-2b-22: -8.6 | TS-2b-3a-12: -23.4 | TS-3a-4c-02: -23.3 | TS-5b-12: -14.5 |
| TS-2c-22: -6.6 | TS-2b-3b-12: -23.2 | TS-3b-4a-02: -23.7 | TS-5c-12: -13.5 |
| | TS-2c-3a-12: -23.0 | TS-3b-4b-02: -25.0 | |
| | TS-2c-3b-12: -20.2 | TS-3b-4c-02: -21.4 | |
| | TS-2c-3c-12: -21.3 | TS-3c-4b-02: -24.8 | |

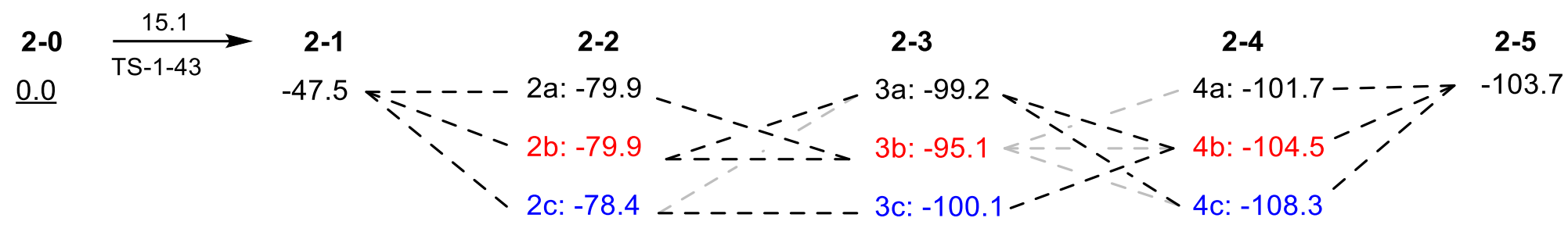


| **2-1** to **2-2** | **2-2** to **2-3** | **2-3** to **2-4** | **2-4** to **2-5** |
|---|---|---|---|
| TS-2a-33: -28.8 | TS-2a-3b-23: -57.0 | TS-3a-4b-13: -71.0 | TS-5a-12: -79.9 |
| TS-2b-33: -28.9 | TS-2b-3a-23: -57.9 | TS-3a-4c-13: -74.4 | TS-5b-12: -76.7 |
| TS-2c-33: -29.3 | TS-2b-3b-23: -58.9 | TS-3b-4a-13: -71.4 | TS-5c-12: -78.7 |
| | TS-2c-3a-23: -56.3 | TS-3b-4b-13: -74.0 | |
| | TS-2c-3b-23: -53.3 | TS-3b-4c-13: -69.6 | |
| | TS-2c-3c-23: -56.4 | TS-3c-4b-13: -74.5 | |

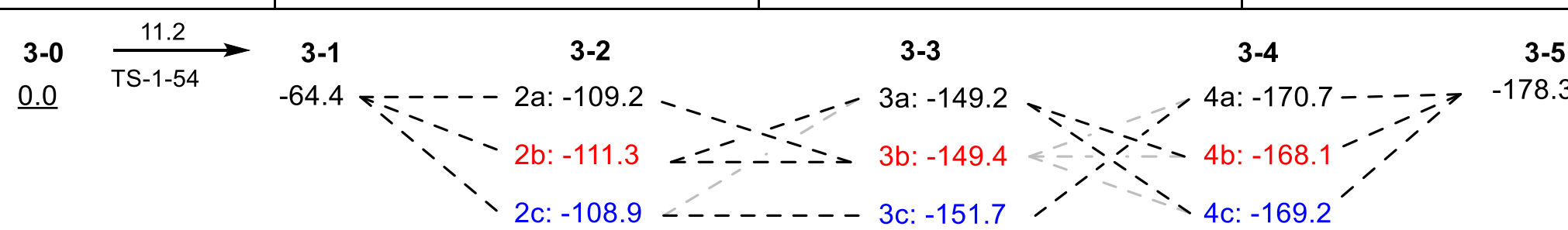


| **3-1** to **3-2** | **3-2** to **3-3** | **3-3** to **3-4** | **3-4** to **3-5** |
|---|---|---|---|
| TS-2a-44: -28.8 | TS-2a-3b-34: -94.1 | TS-3a-4b-24: -126.6 | TS-5a-14: -143.2 |
| TS-2b-44: -28.9 | TS-2b-3a-34: -94.4 | TS-3a-4c-24: -125.0 | TS-5b-14: -143.7 |
| TS-2c-44: -29.3 | TS-2b-3b-34: -97.1 | TS-3b-4a-24: -123.7 | TS-5c-14: -145.6 |
| | TS-2c-3a-34: -93.9 | TS-3b-4b-24: -126.9 | |
| | TS-2c-3b-34: -91.3 | TS-3b-4c-24: -127.0 | |
| | TS-2c-3c-34: -94.5 | TS-3c-4b-24: -129.4 | |

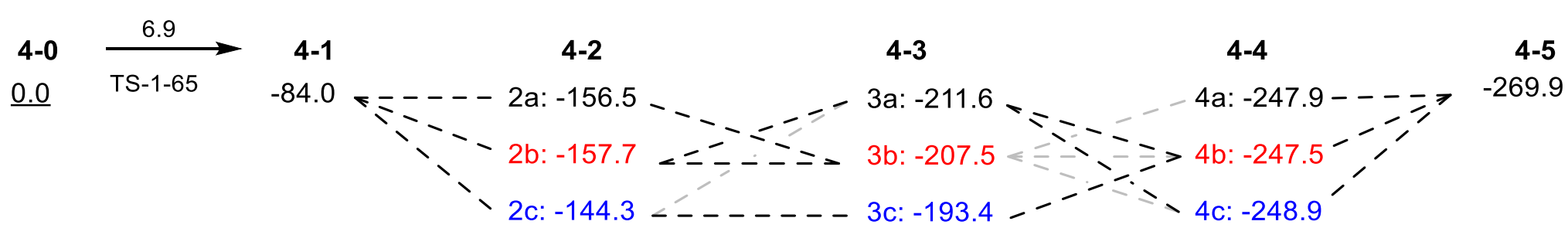


| **4-1** to **4-2** | **4-2** to **4-3** | **4-3** to **4-4** | **4-4** to **4-5** |
|---|---|---|---|
| TS-2a-55: -75.5 | TS-2a-3b-45: -143.9 | TS-3a-4b-35: -193.6 | TS-5a-14: -223.5 |
| TS-2b-55: -71.7 | TS-2b-3a-45: -145.4 | TS-3a-4c-35: -189.7 | TS-5b-14: -226.9 |
| TS-2c-55: -73.0 | TS-2b-3b-45: -142.3 | TS-3b-4a-35: -188.7 | TS-5c-14: -225.9 |
| | TS-2c-3a-45: -139.1 | TS-3b-4b-35: -190.5 | |
| | TS-2c-3b-45: | TS-3b-4c-35: -182.5 | |
| | TS-2c-3c-45: -134.6 | TS-3c-4b-35: -184.2 | |

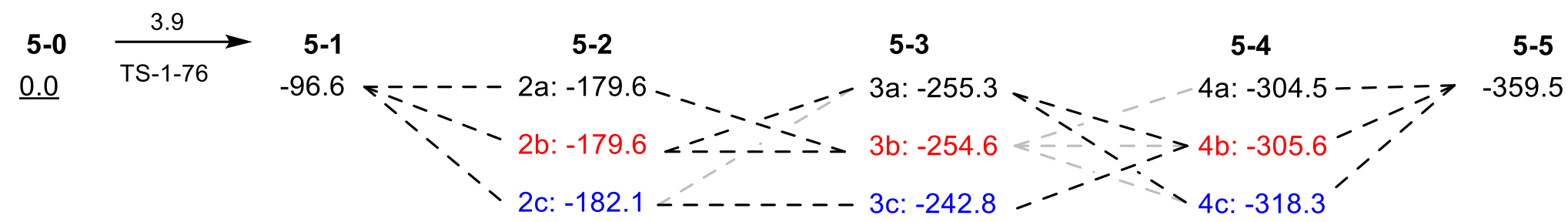


| **5-1** to **5-2** | **5-2** to **5-3** | **5-3** to **5-4** | **5-4** to **5-5** |
|---|---|---|---|
| TS-2a-66: -89.4 | TS-2a-3b-56: -172.6 | TS-3a-4b-46: -241.7 | TS-5a-36: -298.8 |
| TS-2b-66: -90.0 | TS-2b-3a-56: -170.6 | TS-3a-4c-46: -245.0 | TS-5b-36: -294.6 |
| TS-2c-66: -89.9 | TS-2b-3b-56: -172.2 | TS-3b-4a-46: -239.9 | TS-5c-36: -292.8 |
| | TS-2c-3a-56: -173.0 | TS-3b-4b-46: -240.2 | |
| | TS-2c-3b-45: -172.8 | TS-3b-4c-46: -244.8 | |
| | TS-2c-3c-56: -170.9 | TS-3c-4b-46: -293.5 | |

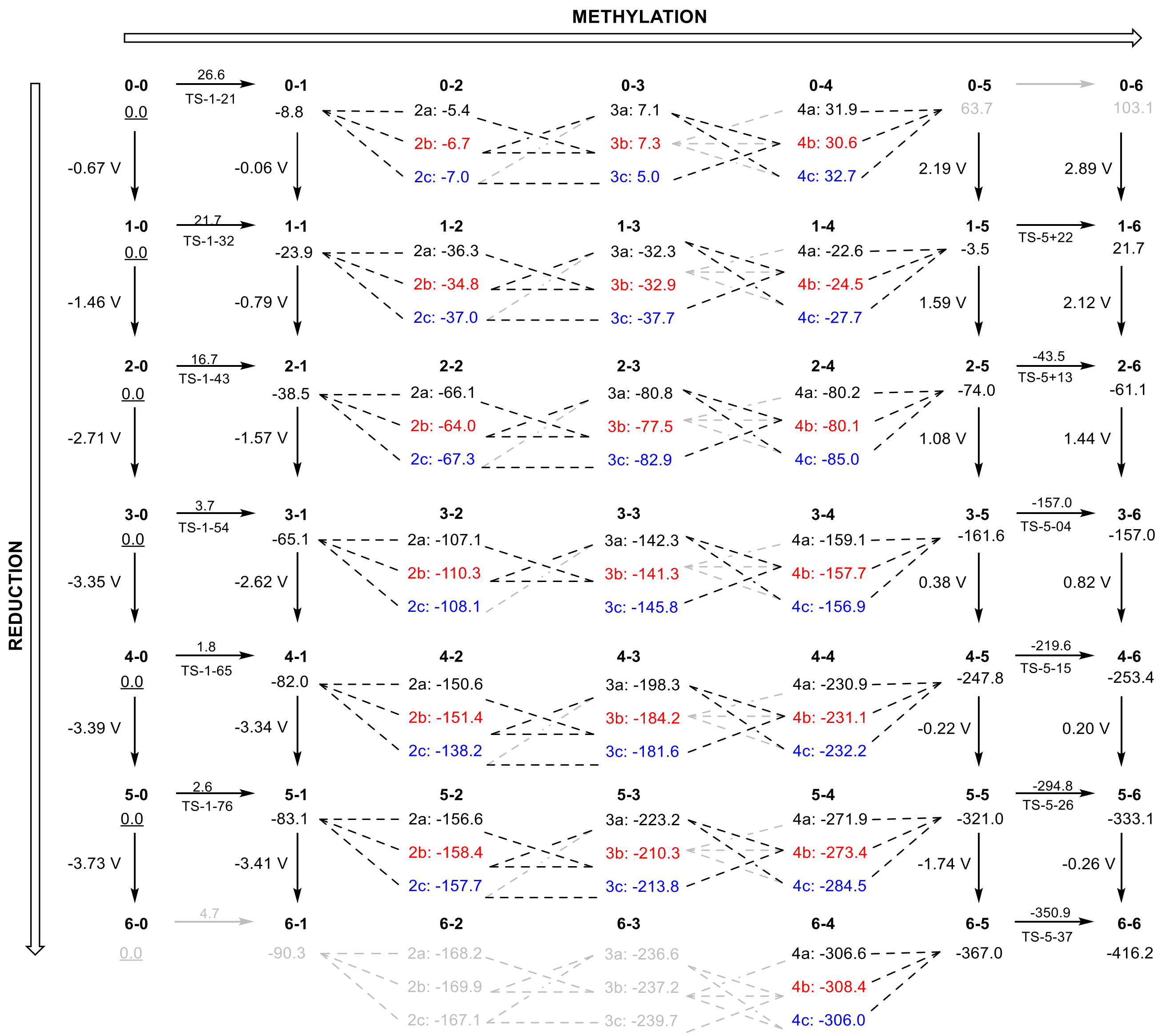


**Figure S3.** Full methylation speciation chemical space starting at $[V_6O_{13}(TRIOL^{NO_2})]^{2-}$cluster, computed at PBE0-D4-SMD(ACN)/BS1 level theory. Gibbs free energies in kcal/mol and reduction potential in Volts respect the $Fc^+/Fc$ reference.

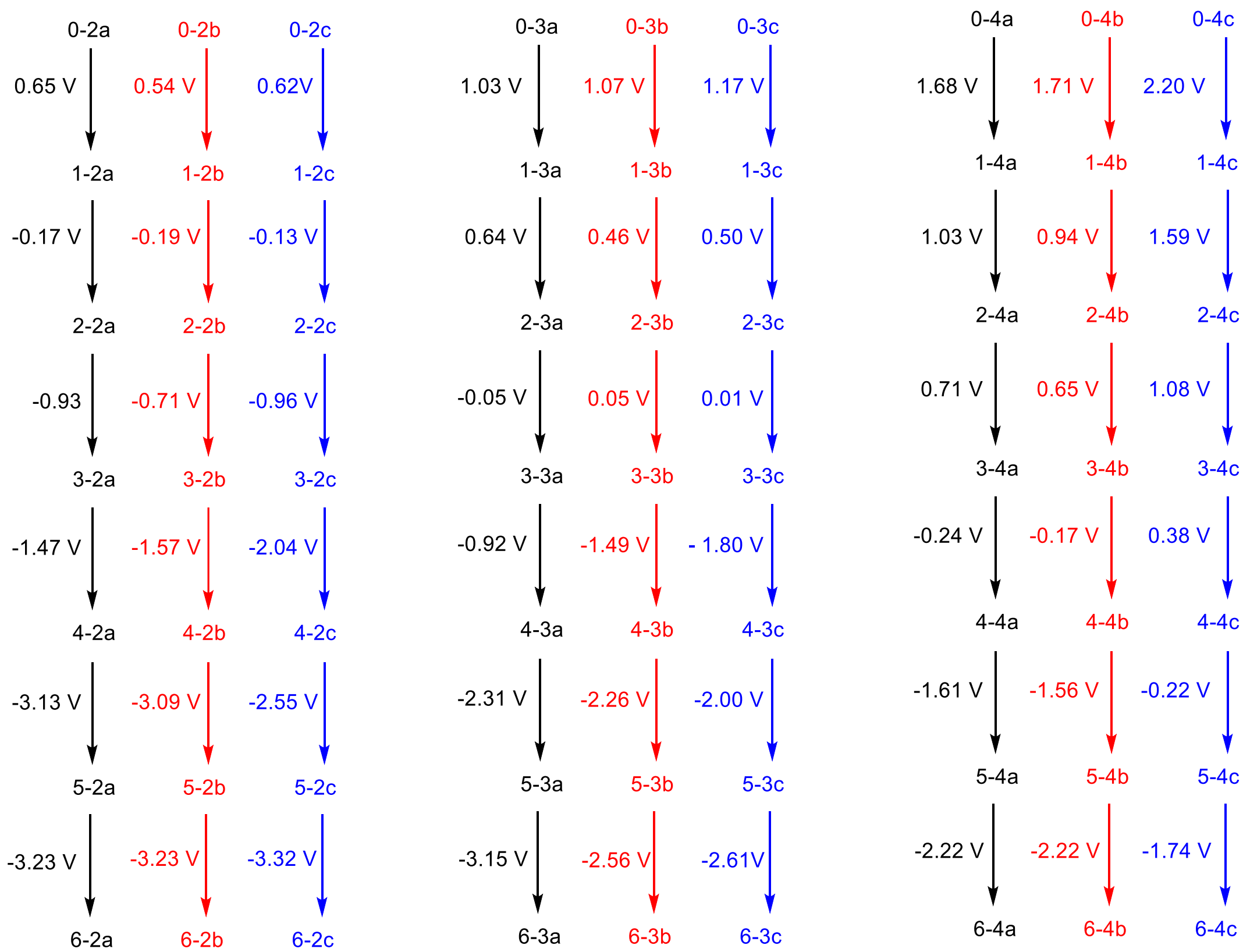


**Figure S4.** Reduction potentials for 0-2, 0-3, and 0-4 intermediates with $TRIOL^{NO_2}$ associated with their isomers, computed at PBE0-D4-SMD(ACN)/BS1 level theory. Reduction potential in Volts respect the $Fc^{+}/Fc$ reference.

**Table S2.** Transition state energetics of methylation $[V_6O_{13}(TRIOL^{NO_2})]^{2-}$ cluster, computed at PBE0-D4-SMD(ACN)/BS1 level theory. Gibbs free energies in kcal/mol.

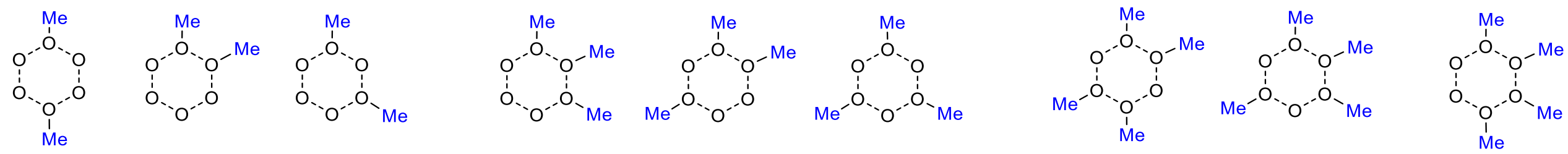


2a-01 2b-01 2c-01 3a+11 3b+11 3c+11 4a+21 4b+21 4c+21

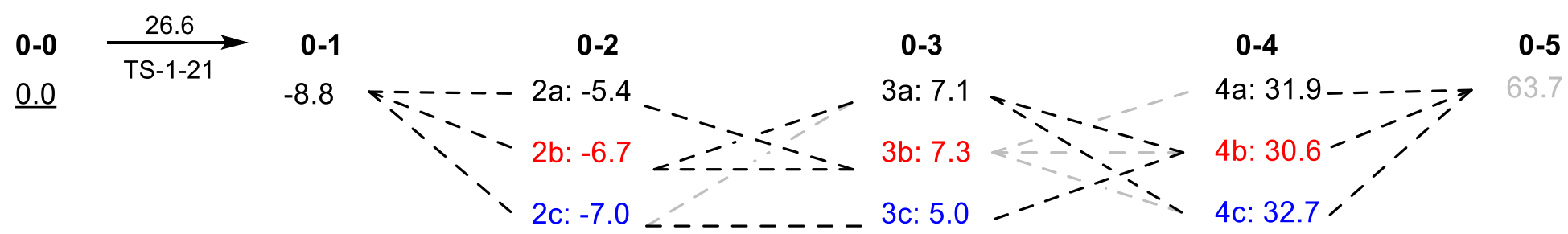


| **0-1** to **0-2** | **0-2** to **0-3** | **0-3** to **0-4** | **0-4** to **0-5** |
|---|---|---|---|
| TS-2a-11: 22.1 | TS-2a-3b-01: 27.7 | TS-3a-4b+11: 48.8 | TS-5a-21: 74.5 |
| TS-2b-11: 21.7 | TS-2b-3a-01: 28.8 | TS-3a-4c+11: 45.1 | TS-5b-21: 73.3 |
| TS-2c-11: 21.2 | TS-2b-3b-01: 28.6 | TS-3b-4a+11: 44.8 | TS-5c-21: 57.4 |
| | TS-2c-3a-01: 27.4 | TS-3b-4b+11: 46.8 | |
| | TS-2c-3b-01: 28.5 | TS-3b-4c+11: 46.2 | |
| | TS-2c-3c-01: 27.3 | TS-3c-4b+11: 45.6 | |

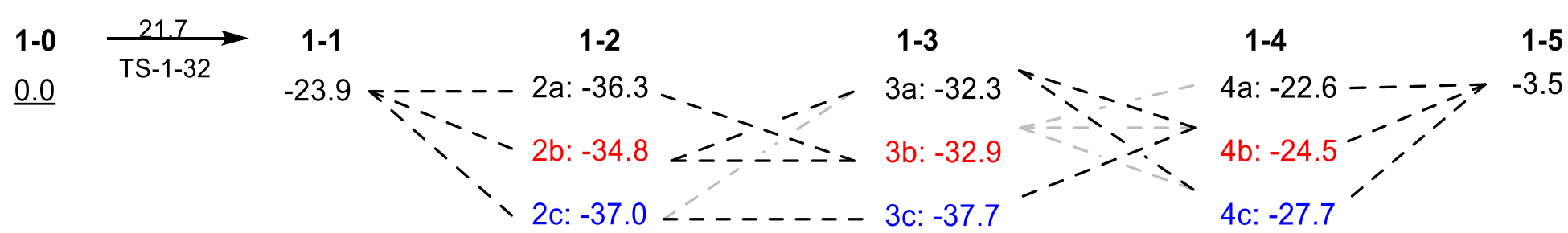


| **1-1** to **1-2** | **1-2** to **1-3** | **1-3** to **1-4** | **1-4** to **1-5** |
|---|---|---|---|
| TS-2a-22: 0.3 | TS-2a-3b-12: -7.6 | TS-3a-4b-02: -5.1 | TS-5a-12: 10.8 |
| TS-2b-22: -1.3 | TS-2b-3a-12: -10.6 | TS-3a-4c-02: -6.3 | TS-5b-12: 10.6 |
| TS-2c-22: 0.9 | TS-2b-3b-12: -10.0 | TS-3b-4a-02: -5.8 | TS-5c-12: 10.8 |
| | TS-2c-3a-12: -9.8 | TS-3b-4b-02: -6.0 | |
| | TS-2c-3b-12: -7.4 | TS-3b-4c-02: -3.1 | |
| | TS-2c-3c-12: -8.1 | TS-3c-4b-02: -3.7 | |

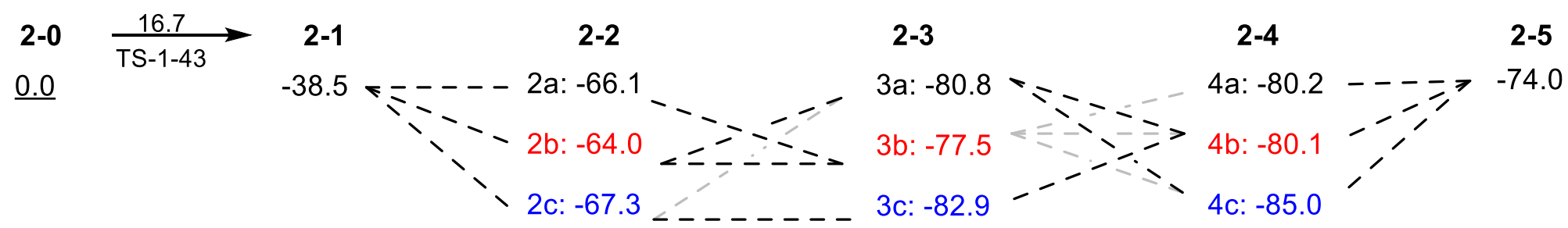


| **2-1** to **2-2** | **2-2** to **2-3** | **2-3** to **2-4** | **2-4** to **2-5** |
|---|---|---|---|
| TS-2a-33: -21.4 | TS-2a-3b-23: -41.7 | TS-3a-4b-13: -53.5 | TS-5a-12: -68.2 |
| TS-2b-33: -22.2 | TS-2b-3a-23: -42.5 | TS-3a-4c-13: -55.9 | TS-5b-12: -54.9 |
| TS-2c-33: -20.6 | TS-2b-3b-23: -44.4 | TS-3b-4a-13: -56.0 | TS-5c-12: -50.5 |
| | TS-2c-3a-23: -41.7 | TS-3b-4b-13: -54.6 | |
| | TS-2c-3b-23: -42.5 | TS-3b-4c-13: -57.4 | |
| | TS-2c-3c-23: -44.4 | TS-3c-4b-13: -53.2 | |

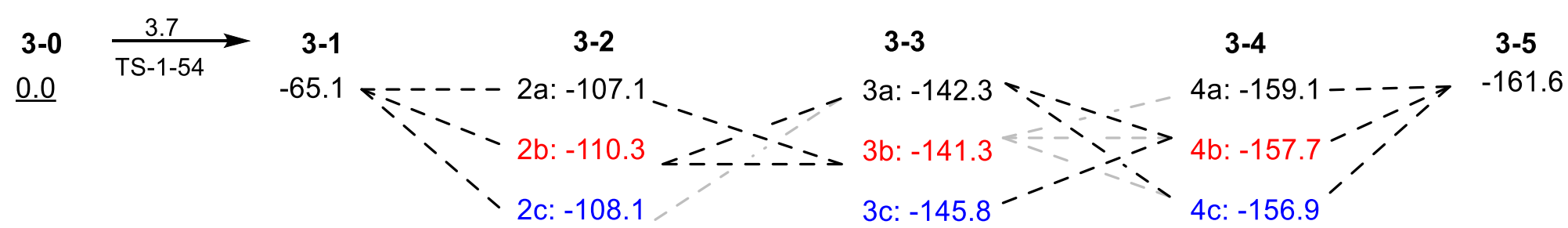


| **3-1** to **3-2** | **3-2** to **3-3** | **3-3** to **3-4** | **3-4** to **3-5** |
|---|---|---|---|
| TS-2a-44: -50.5 | TS-2a-3b-34: -90.9 | TS-3a-4b-24: -118.5 | TS-5a-14: -130.5 |
| TS-2b-44: -45.1 | TS-2b-3a-34: -92.5 | TS-3a-4c-24: -117.6 | TS-5b-14: -132.1 |
| TS-2c-44: -52.1 | TS-2b-3b-34: -93.9 | TS-3b-4a-24: -121.3 | TS-5c-14: -131.7 |
| | TS-2c-3a-34: -91.6 | TS-3b-4b-24: -119.6 | |
| | TS-2c-3b-34: -91.3 | TS-3b-4c-24: -118.2 | |
| | TS-2c-3c-34: -91.8 | TS-3c-4b-24: -117.5 | |

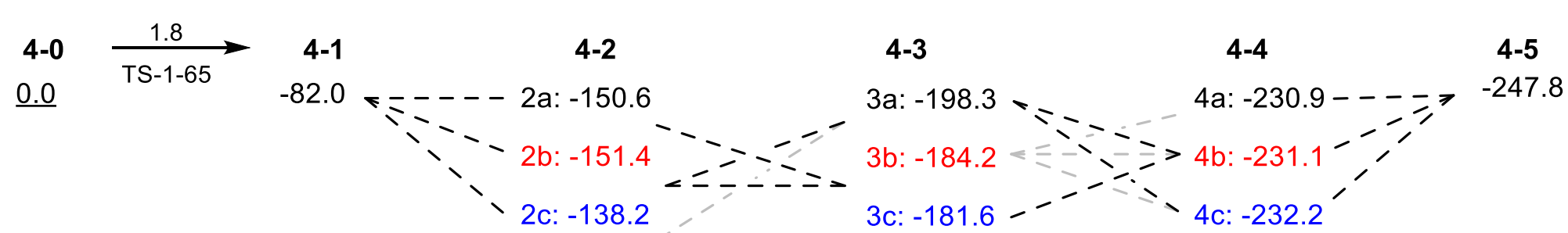


| **4-1** to **4-2** | **4-2** to **4-3** | **4-3** to **4-4** | **4-4** to **4-5** |
|---|---|---|---|
| TS-2a-55: -70.6 | TS-2a-3b-45: -148.7 | TS-3a-4b-35: -210.2 | TS-5a-14: -264.1 |
| TS-2b-55: -64.7 | TS-2b-3a-45: -148.4 | TS-3a-4c-35: -210.7 | TS-5b-14: -258.4 |
| TS-2c-55: -69.5 | TS-2b-3b-45: -147.0 | TS-3b-4a-35: -202.3 | TS-5c-14: -258.4 |
| | TS-2c-3a-45: -147.5 | TS-3b-4b-35: -209.8 | |
| | TS-2c-3b-45: -147.1 | TS-3b-4c-35: -213.8 | |
| | TS-2c-3c-45: -145.7 | TS-3c-4b-35: -212.1 | |

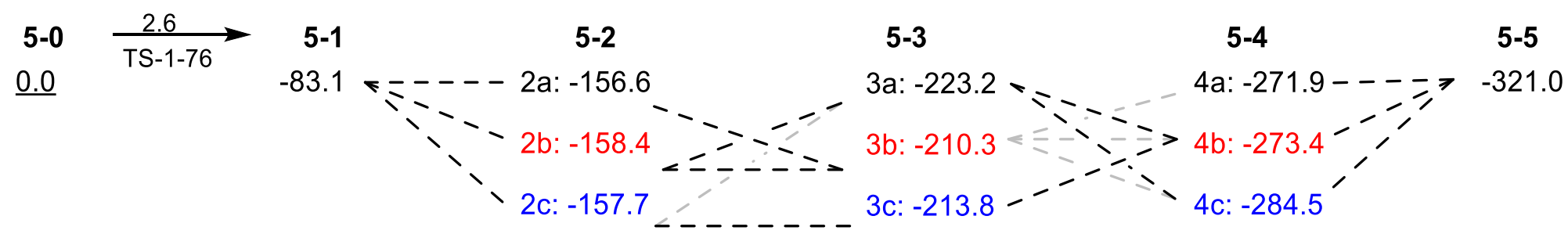


| **5-1** to **5-2** | **5-2** to **5-3** | **5-3** to **5-4** | **5-4** to **5-5** |
|---|---|---|---|
| TS-2a-66: -83.0 | TS-2a-3b-56: -222.9 | TS-3a-4b-46: -224.5 | TS-5a-36: -283.4 |
| TS-2b-66: -83.4 | TS-2b-3a-56: -155.4 | TS-3a-4c-46: -225.2 | TS-5b-36: -297.1 |
| TS-2c-66: -80.1 | TS-2b-3b-56: -156.9 | TS-3b-4a-46: -231.1 | TS-5c-36: -296.2 |
| | TS-2c-3a-56: -157.4 | TS-3b-4b-46: -224.7 | |
| | TS-2c-3b-45: -156.1 | TS-3b-4c-46: -227.7 | |
| | TS-2c-3c-56: -155.5 | TS-3c-4b-46: -227.4 | |

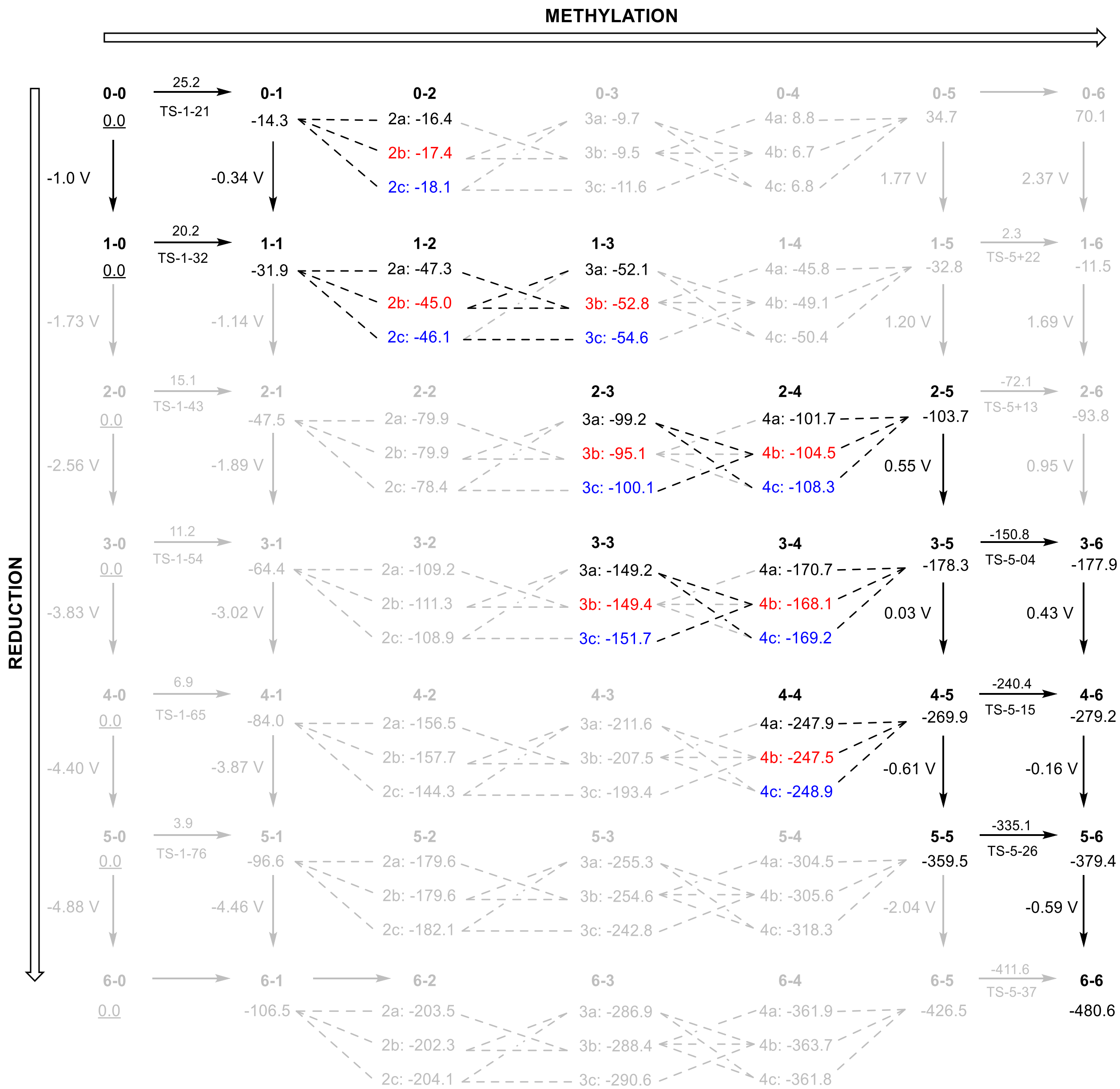


**Figure S5.** Full methylation and reduction speciation chemical space starting at $[V_6O_{13}(TRIOL^{Me})_2]^{2-}$cluster, highlighting the favorable steps that remain visible under conditions with reduction potentials E < –1.0 V vs $Fc^+$/Fc and activation barriers $\Delta^{\ddagger}G$ > 29 kcal/mol.

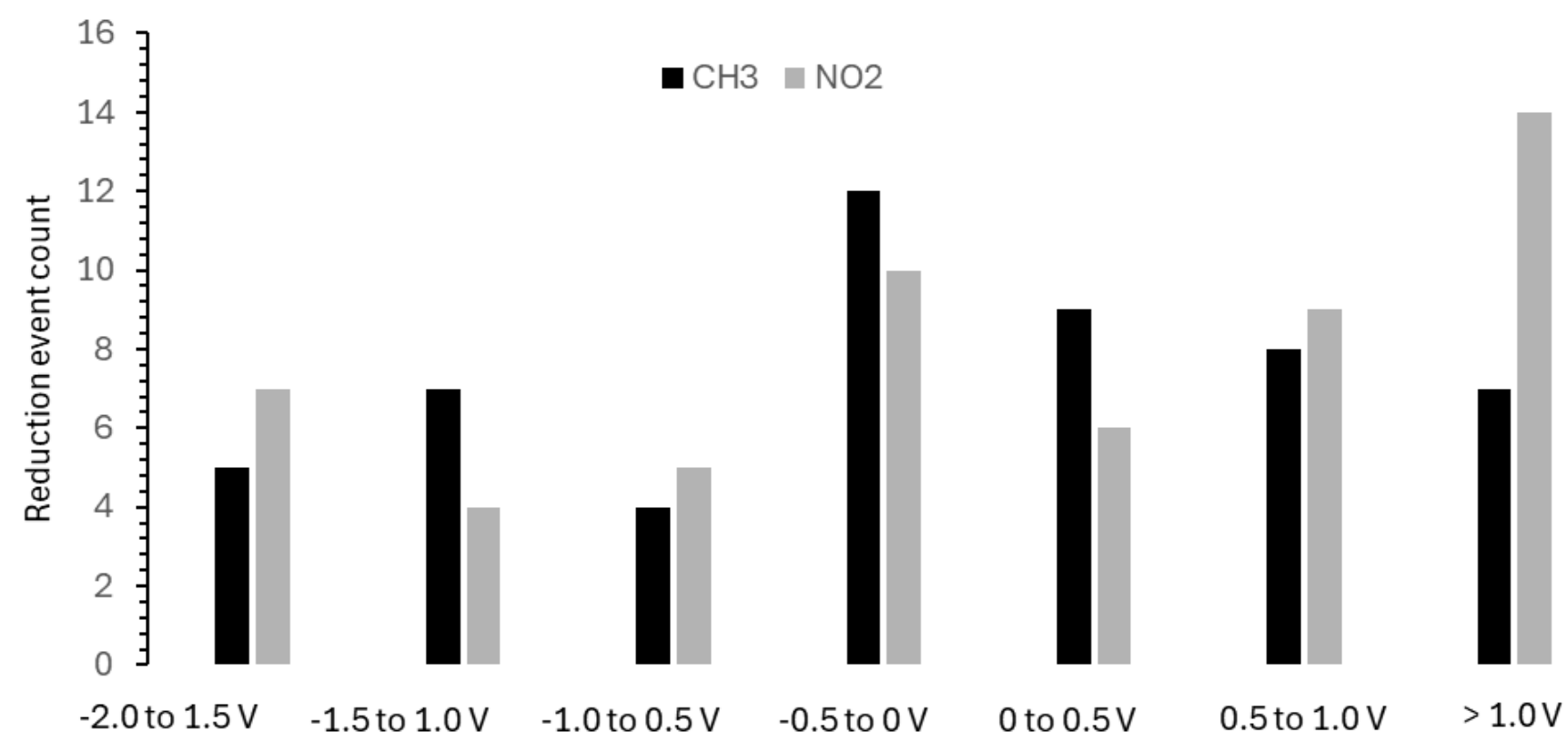


**Figure S6.** Distribution of the reduction potentials of TRIOL$^{X}$-substituted clusters (X = $-CH_3$ and $-NO_2$) across studied chemical space.

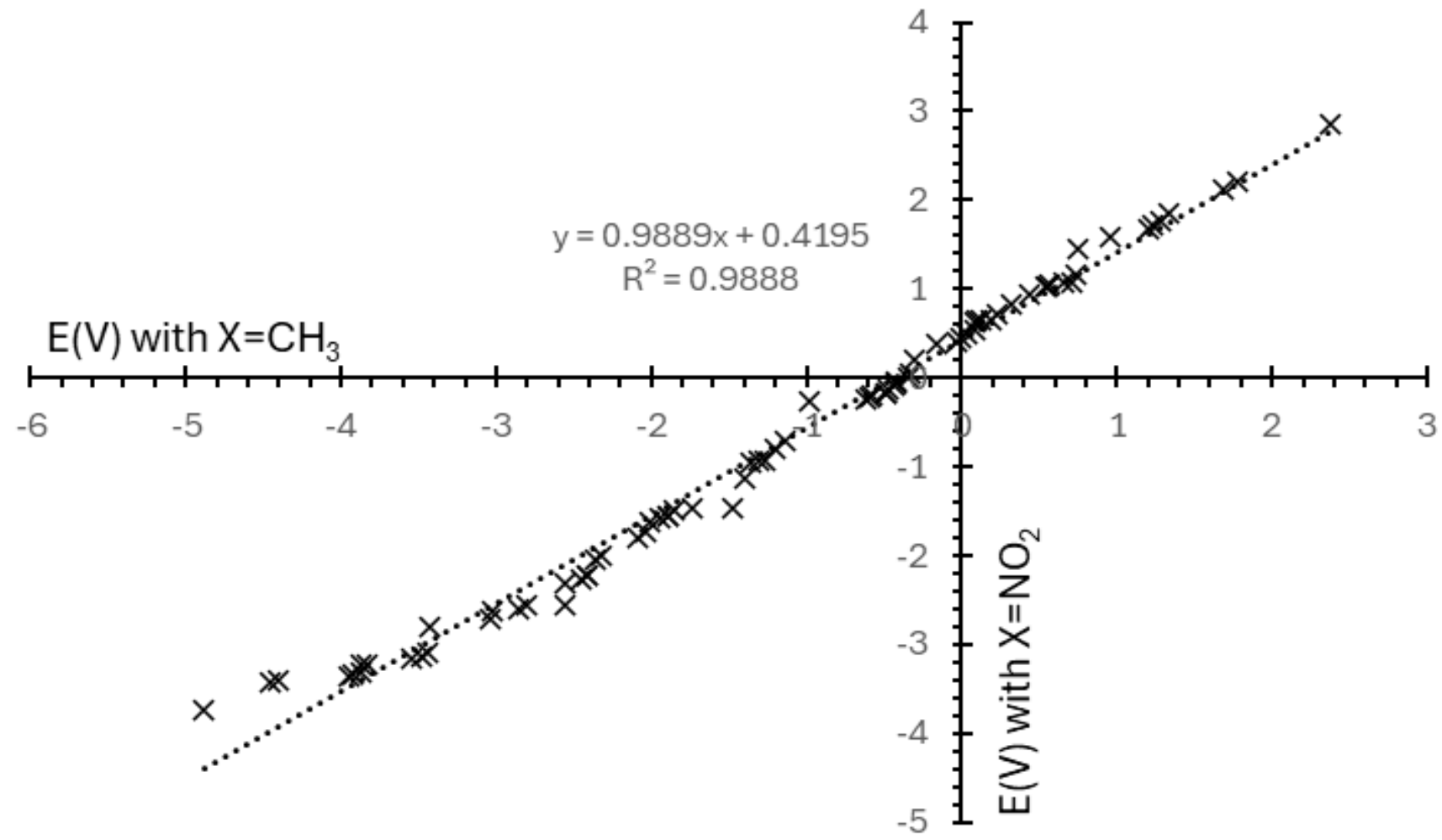


**Figure S7.** Correlation between the redox potentials of TRIOL$^X$-substituted clusters with X = $-CH_3$ and $-NO_2$.

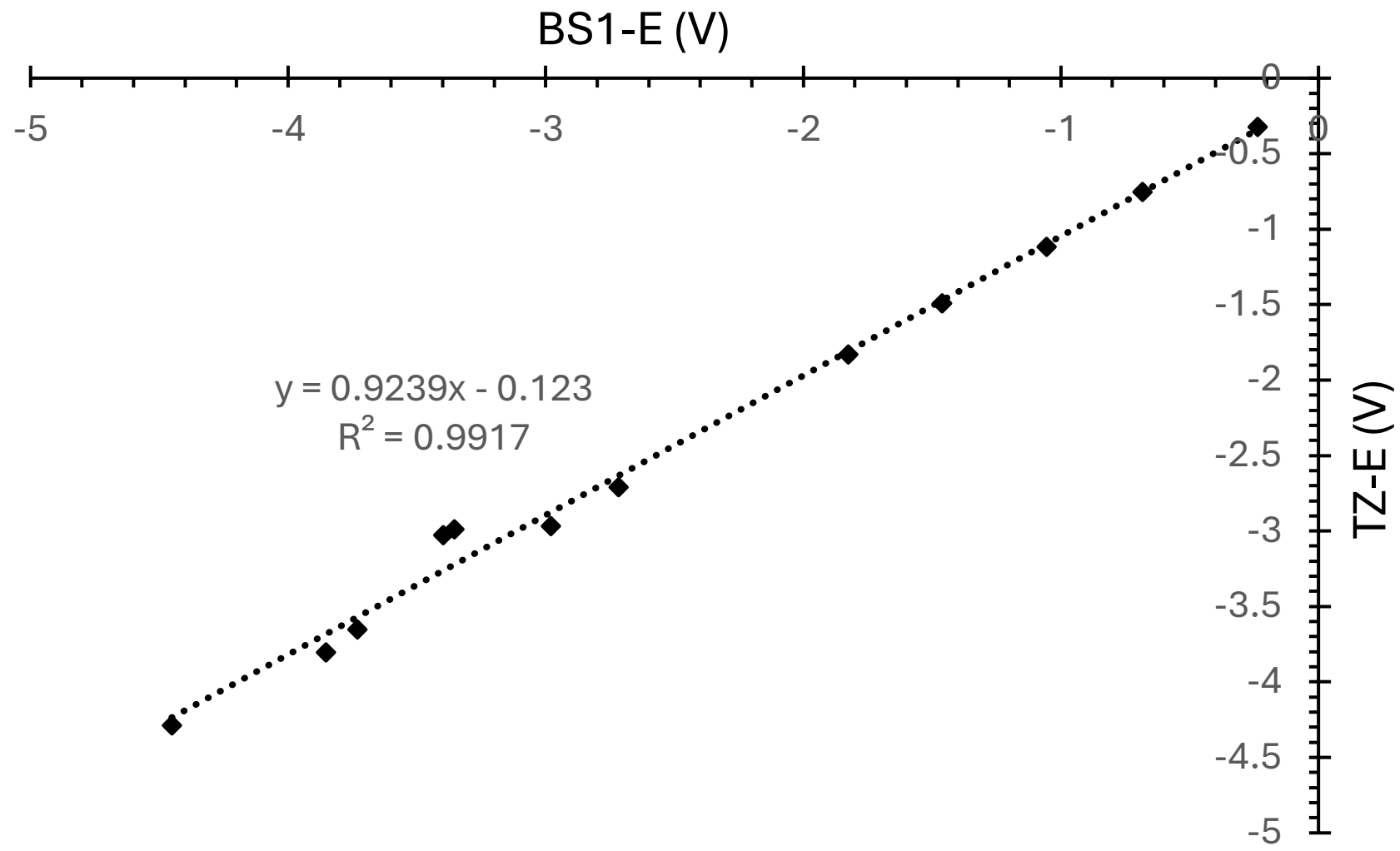


**Figure S8.** The correlation of reduction potential correlation calculated SMD(THF)-PBE0-D4/BS1 and SMD(THF)-PBE0-D4/def2-TZVP//BS1 level of theory. The data for this plot is tabulated in Table S7.

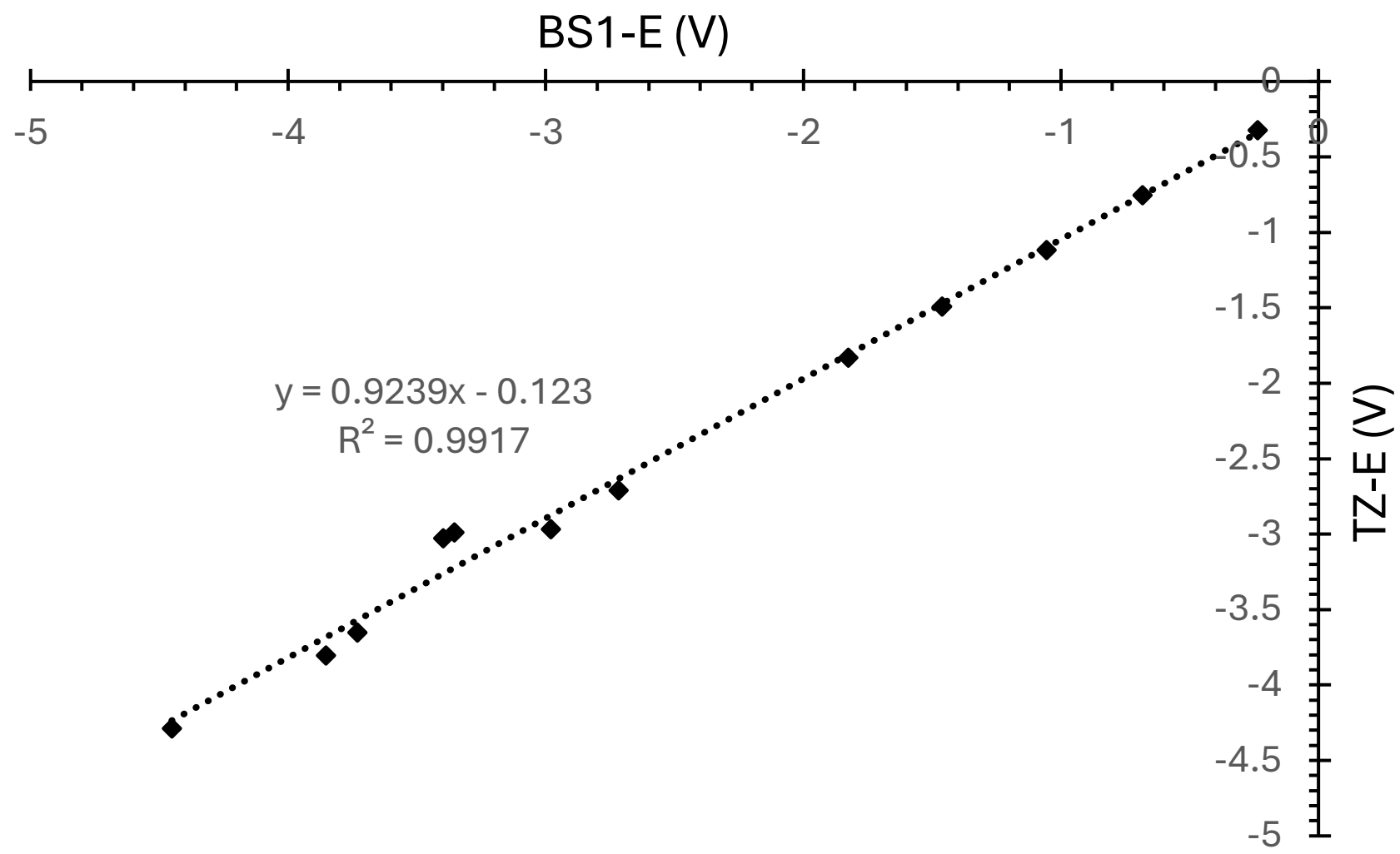


**Figure S9.** The correlation of reduction potential correlation calculated SMD(THF)-PBE0-D4/BS1 and SMD(THF)-PBE0-D4/def2-TZVP//BS1 level of theory. The data for this plot is tabulated in Table S7.

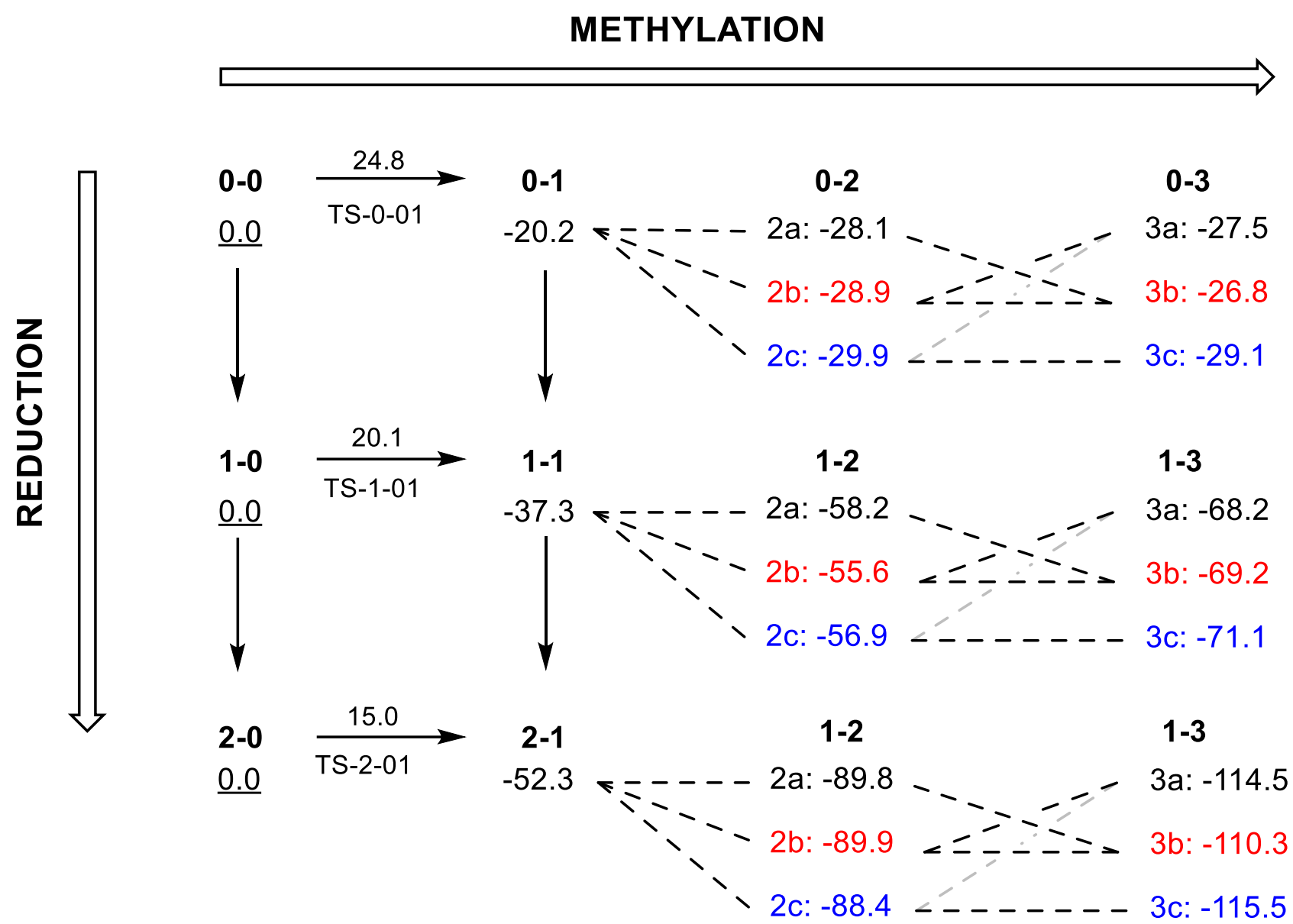


**Figure S10.** Energetic profile calculated at SMD(THF)-PBE0-D4/def2-TZVP//SMD(THF)-PBE0-D4/BS1 level theory. This scheme is used for the microkinetic modeling presented in Figure 4

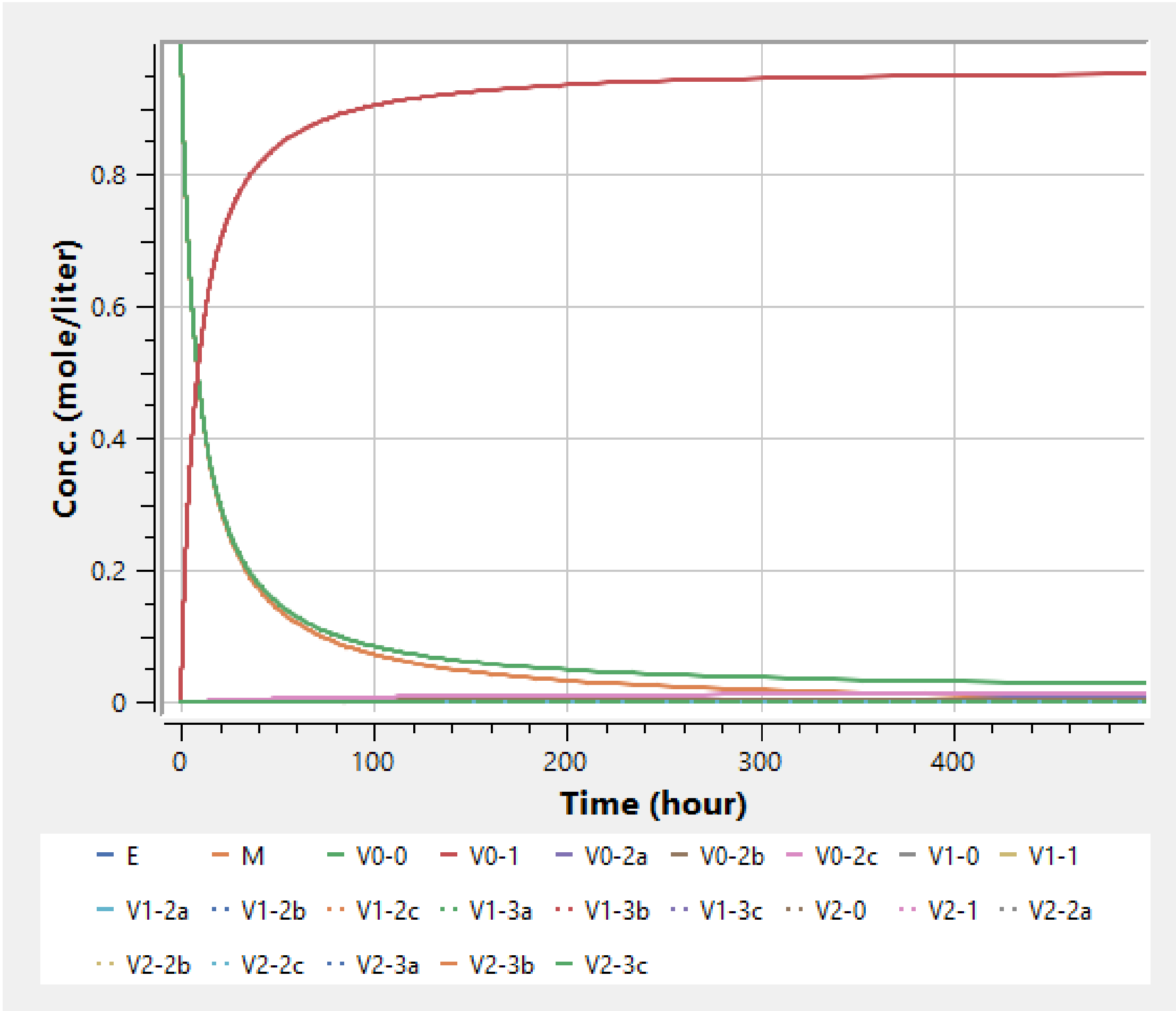


**Figure S11.** The microkinetic modelling results for the subspace presented in Figure 5. Ratio of reducing agent, MeOTf, and $[V^{V}_{6}O_{13}(TRIOL^{X})_{2}]^{2-}$ was set to 0:1:1.

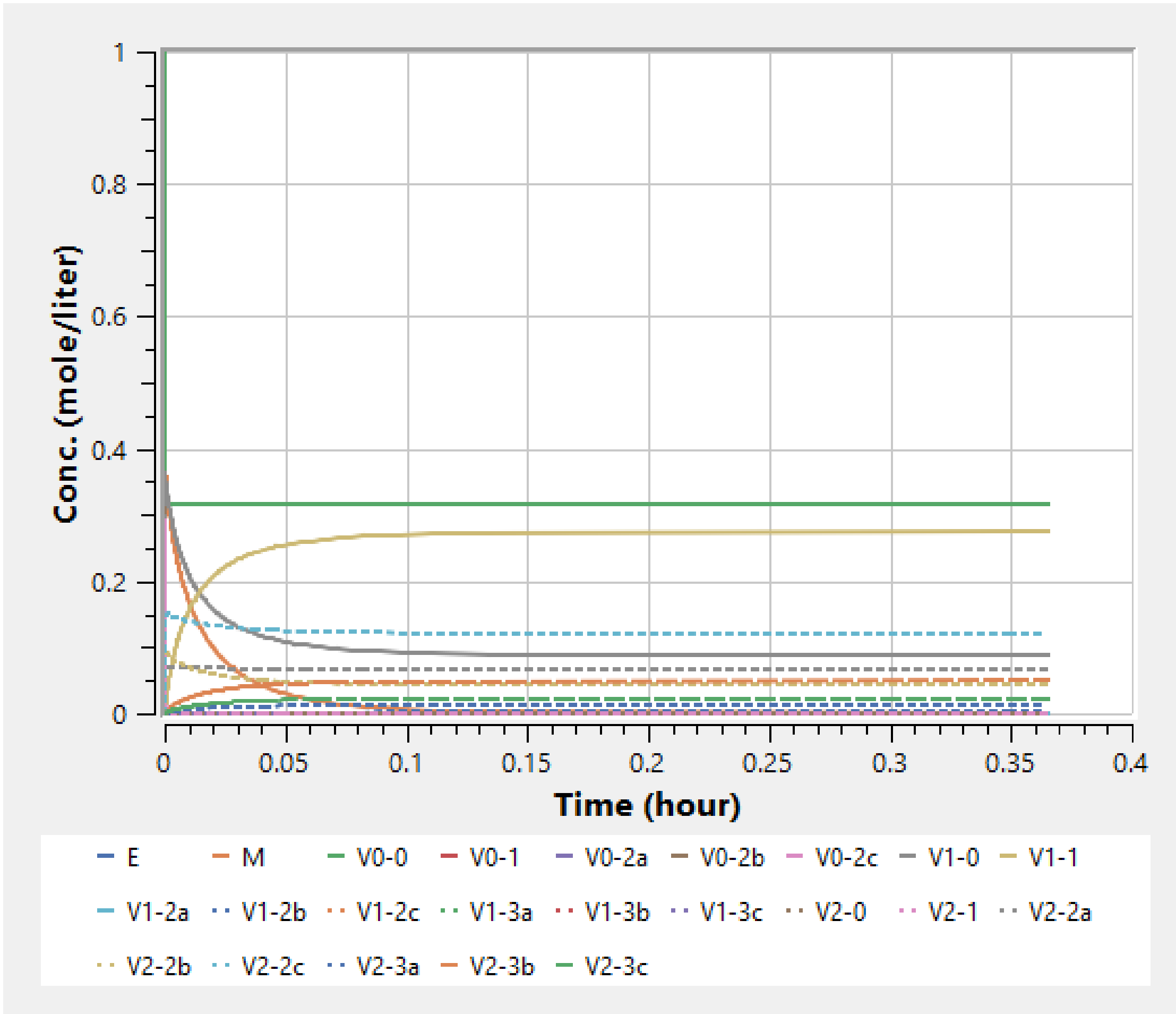


**Figure S12**. The microkinetic modelling results for the subspace presented in Figure 5. Ratio of reducing agent, MeOTf, and $[V^{V}_{6}O_{13}(TRIOL^{X})_2]^{2-}$ was set to 1:1:1.

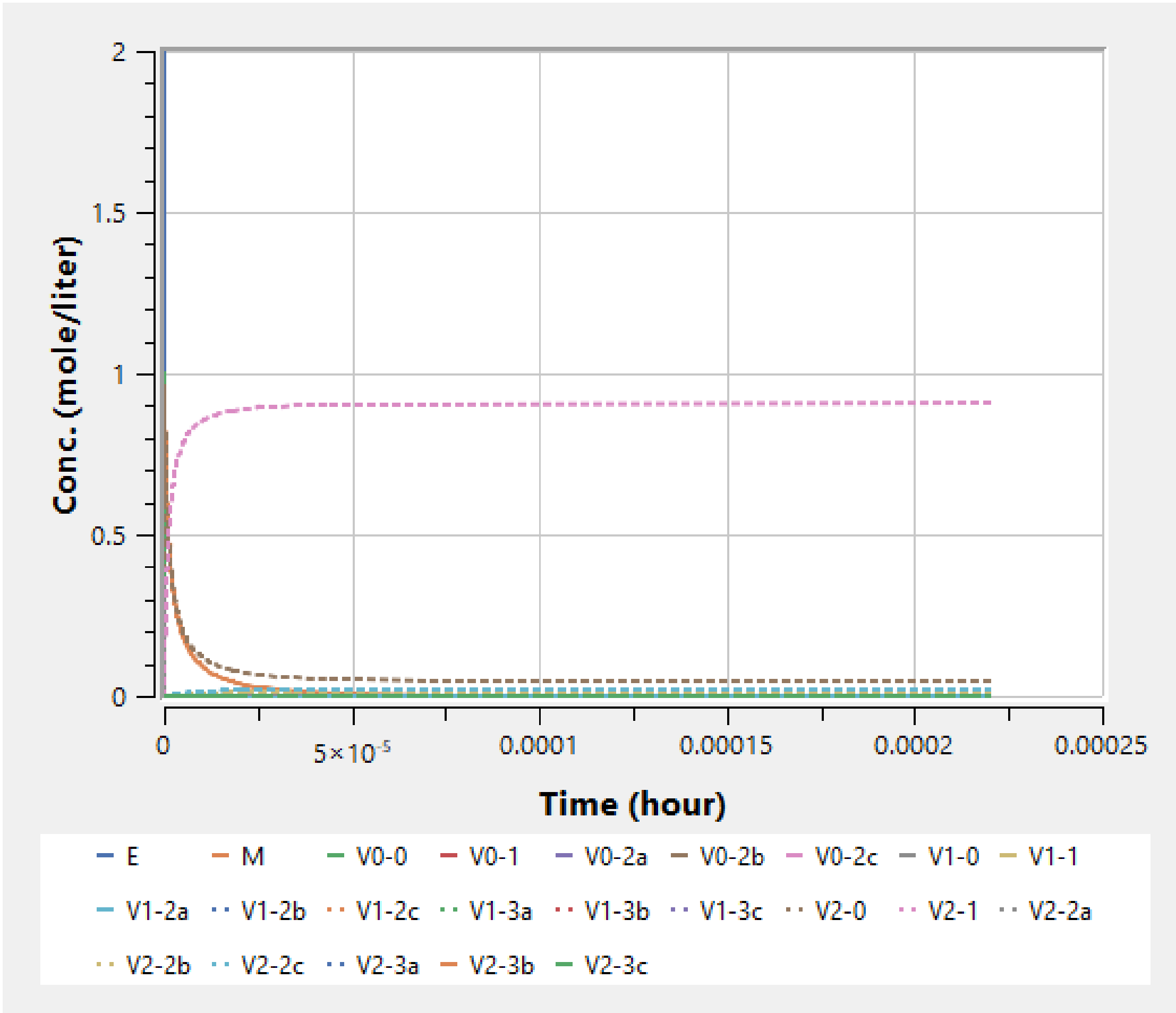


**Figure S13.** The microkinetic modelling results for the subspace presented in Figure 5. Ratio of reducing agent, MeOTf, and $[V^{V}{}_6O_{13}(TRIOL^X)_2]^{2-}$ was set to 2:1:1.

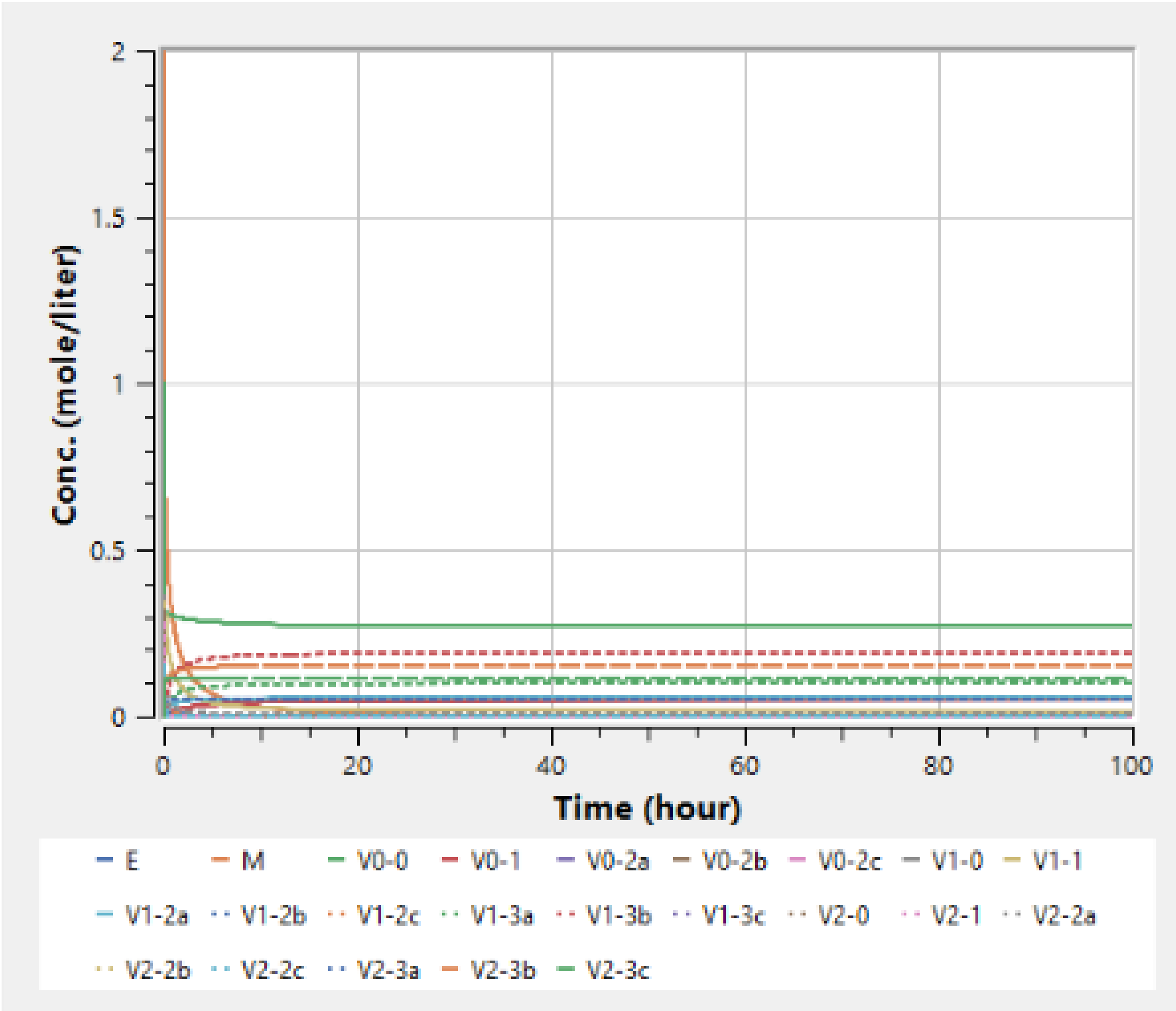


**Figure S14.** The microkinetic modelling results for the subspace presented in Figure 5. Ratio of reducing agent, MeOTf, and $[V^{V}{}_{6}O_{13}(TRIOL^{X})_{2}]^{2-}$ was set to 1:2:1.

**Table S3.** Computed thermodynamics for the first methylation step of various substituents, calculated at the SMD(THF)-PBE0-D4/BS1 level of theory.

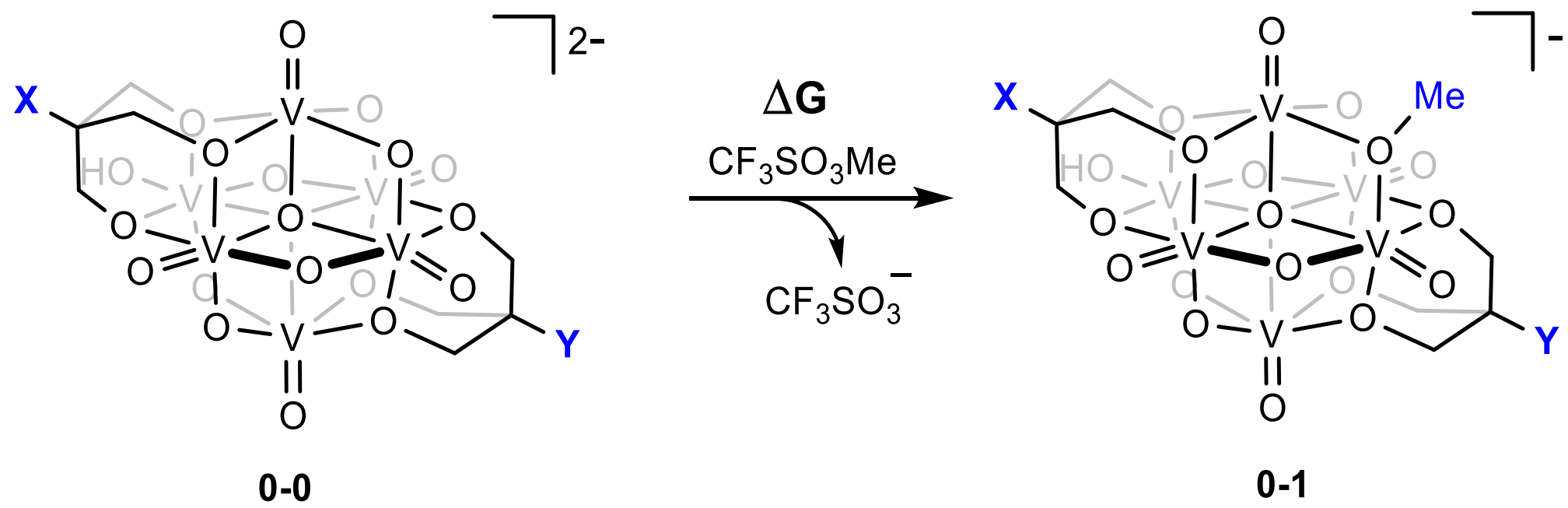


| Substituents (X, Y) | σ | ΔG(kcal/mol) |
|---|---|---|
| X= H, Y= $NO_2$ | 0.76 | -11.58 |
| X= $NO_2$, Y= $NO_2$ | 1.52 | -8.83 |
| X= Me, Y= Me | -0.08 | -14.30 |
| X= Me, Y= $NO_2$ | 0.72 | -11.53 |
| X= H, Y= H | 0.0 | -14.94 |
| X= H, Y= Br | 1.26 | -9.20 |

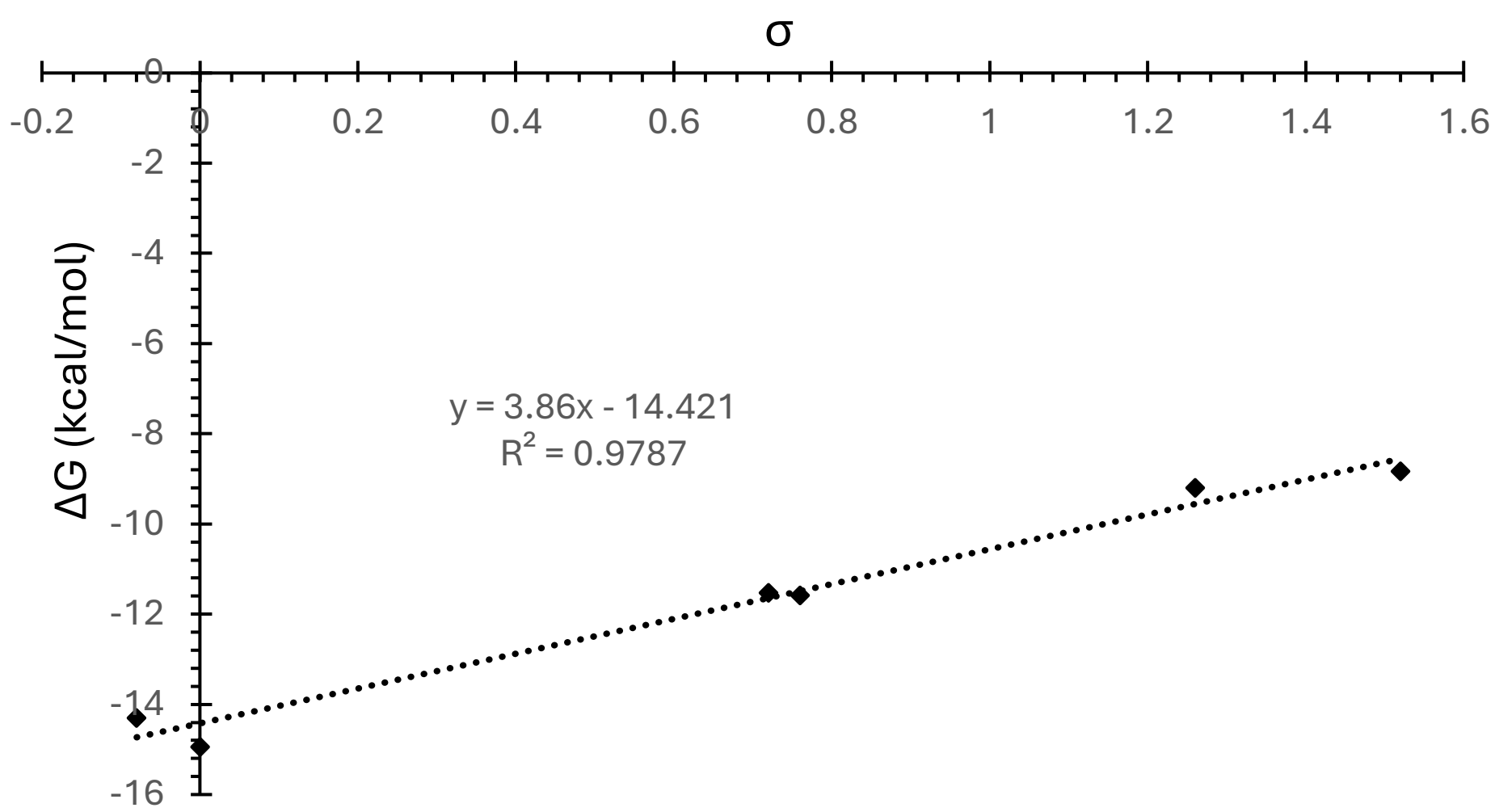


**Figure S15.** Correlation between Hammett parameters and thermodynamic methylation of complex 0-0 and its derivatives calculated at the SMD(THF)-PBE0-D4/BS1 level of theory.

**Table S4.** Computed kinetics for the first methylation step of various substituents, calculated at the SMD(THF)-PBE0-D4/BS1 level of theory.

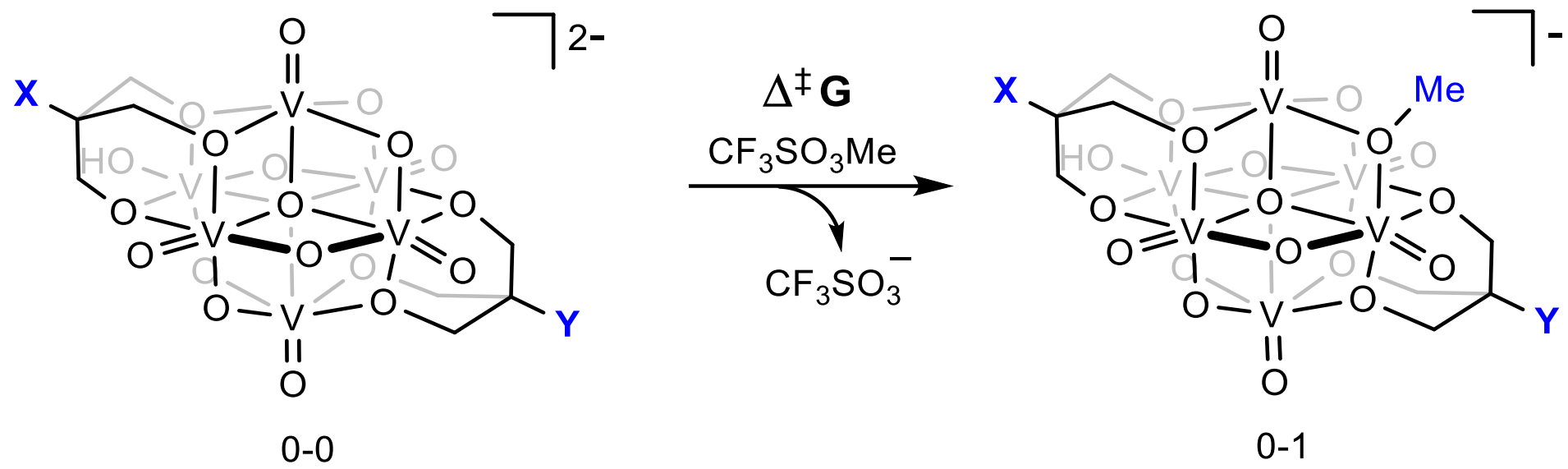


| Substituents (X, Y) | $\sigma = \sigma_X + \sigma_Y$ | BS1-ΔG(kcal/mol) | TZ-ΔG(kcal/mol |
|---|---|---|---|
| X= Cl, Y= Cl | 0.94 | 25.9 | 26.3 |
| X= $NO_2$, Y= $NO_2$ | 1.52 | 26.4 | 26.6 |
| X= Br, Y= Br | 1 | 25.9 | 26.4 |
| X= H, Y= H | -0.08 | 24.8 | 25.2 |
| X= Me, Y= Me | 0.72 | 25.8 | 25.8 |
| X= H, Y= $NO_2$ | 0.76 | 25.7 | 25.9 |

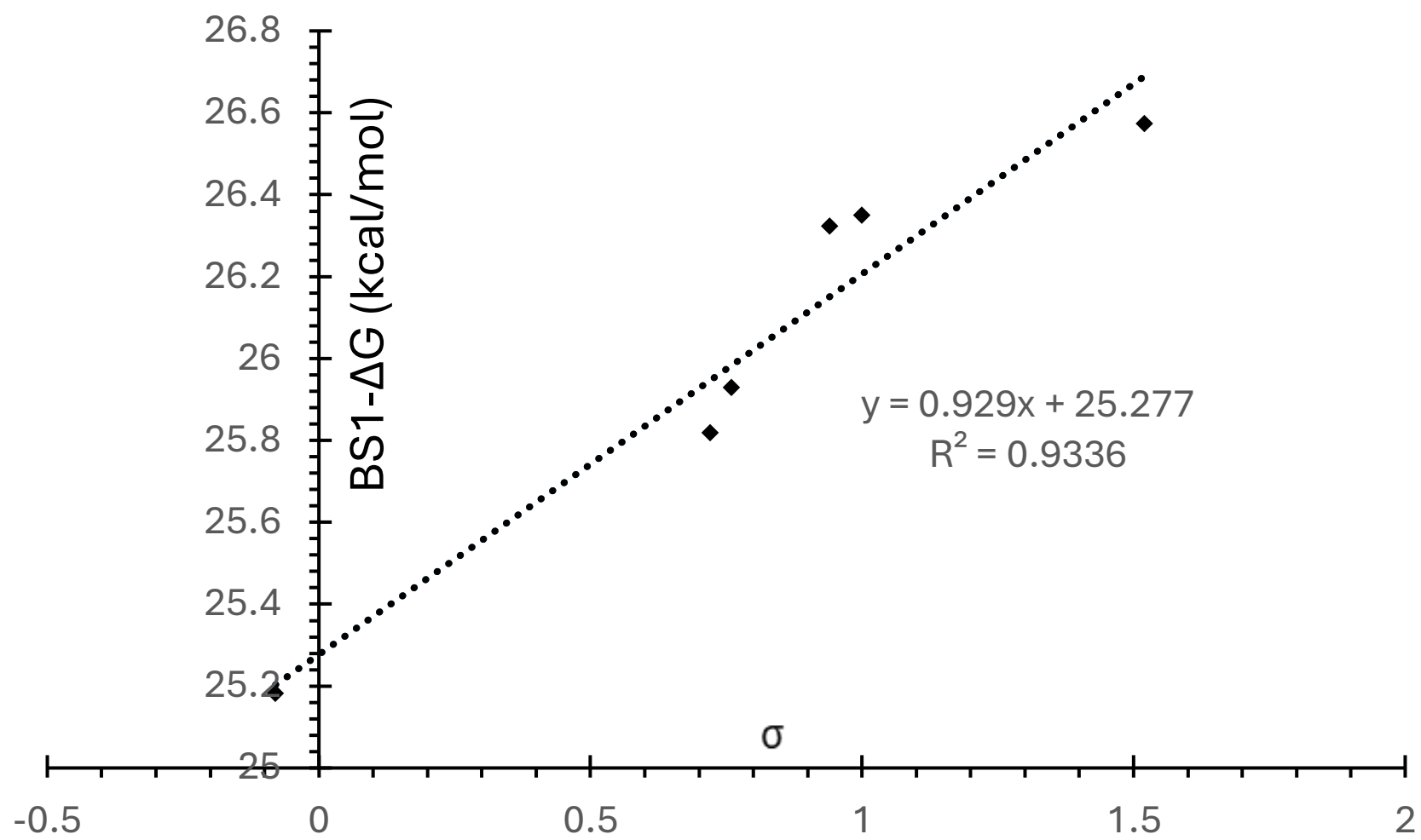


**Figure S16**. Correlation between Hammett parameters and kinetics methylation of compound 0-0 and its derivatives calculated at the SMD(THF)-PBE0-D4/BS1 level of theory.

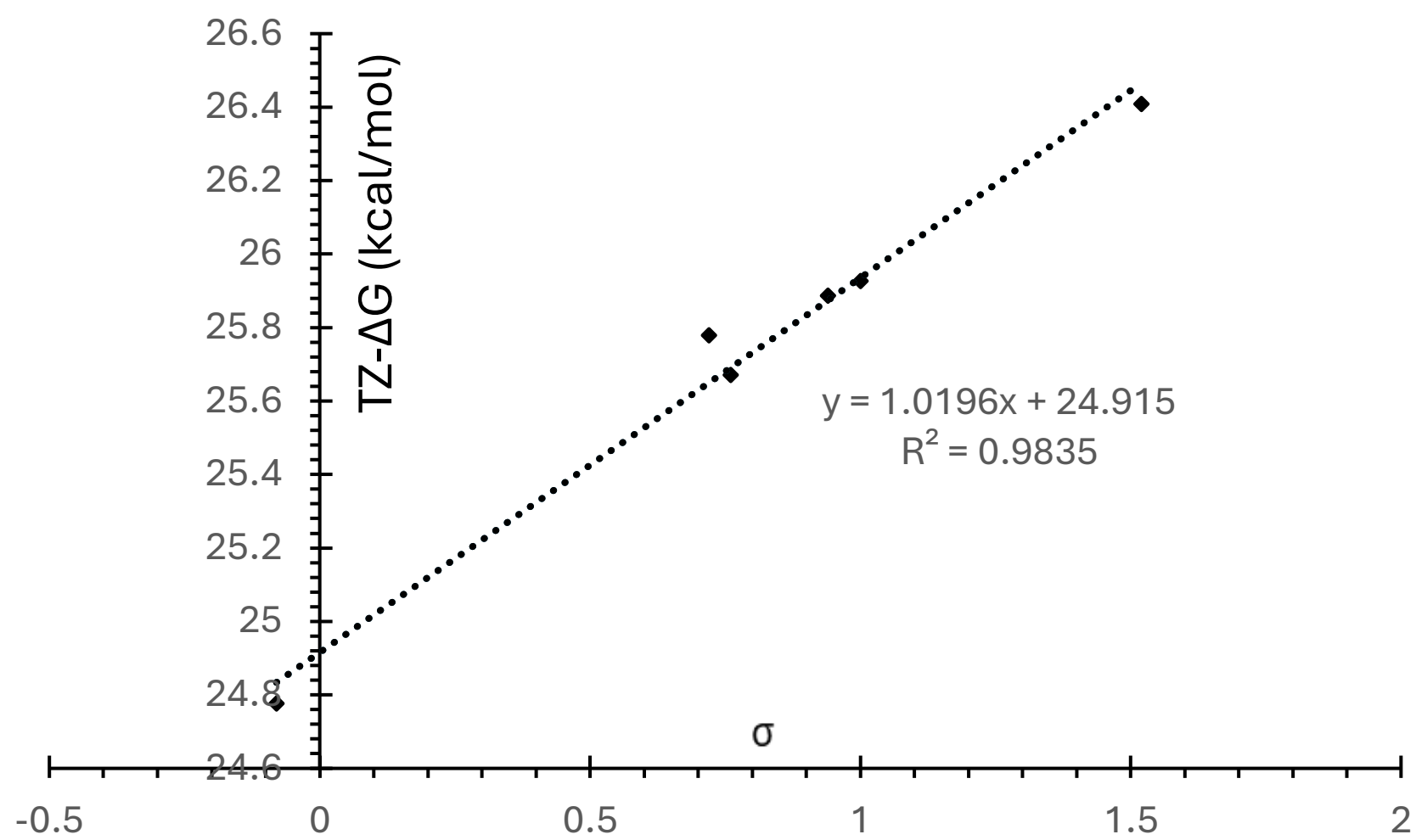


**Figure S17.** Correlation between Hammett parameters and kinetics methylation of compound 0-0 and its derivatives calculated at the the SMD(THF)-PBE0-D4/def2-TZVP//BS1 level of theory.

**S.1 Basis Set Effect**

We examined the effect of the basis set into the speciation thermodynamics and kinetics of the studied chemical space. To our surprise, for each methylation step, the relative energies calculated using the BS1(def2-TZVP for V and S and def-SVP) or denote as SMD(THF)-PBE0-D4//BS1 compared to single point correction at def2-TZVP basis sets for all atoms, the become more and more difference between the two basis sets becomes increasingly pronounced. We attribute this discrepancy to how each basis set treats the separated ionic species that are formed during each methylation step and accumulate in the more highly methylated compounds. To obtain a broader statistical perspective, this procedure was applied to various reduced compounds, and the thermodynamic results are compiled in Table S5. Overall, the larger basis set predicts a more exergonic process compared to the smaller basis set, suggesting a more favorable reaction profile. Despite differences in absolute free energies obtained with two basis sets, the results from each methylation step across different reduced compounds exhibit strong linear correlations ($R^2 > 0.99$) (Figures S19–S21). These consistent trends across both methods support our assumption regarding the systematic influence of basis set choice on the energetics of ion-separated systems.

Linear regression analysis further shows that single-point corrections at the TZ level shift the BS1-level relative free energies by approximately −6.6, −12.5, and −18.2 kcal/mol for the first, second, and third methylation steps, respectively. For additional confirmation, we expected minimal deviation between the two basis sets in stages prior to the completion of the first methylation, specifically, before any ion-pair dissociation occurs. The transition state data for the first methylation step, summarized in **Table S6**, supports this expectation: the energy difference between the two basis sets is less than 0.5 kcal/mol. In contrast, larger deviations in transition states after ion-pair dissociation aligned with these trends. The overall reduction free energies display minor differences with a strong linear correlation ($R^2 = 0.99$, **Figure S8**).

Unlike wavefunction methods, DFT errors are mainly functional-driven and do not systematically improve with larger basis sets. Both BS1 and TZ basis sets provide consistent data trend. Given the large number of geometries, our discussion focuses on BS1 results, while microkinetic modeling incorporates TZ-level single-point corrections, as kinetics such as the rate constant is sensitive with the method of choice.

**Table S5.** Thermodynamic energetics of the methylation of the $[V_6O_{13}(TRIOL^{Me})]^{2-}$ cluster, from compound **0-0** to compound **0-3**, computed at PBE0-D4-SMD(ACN)/BS1 level theory

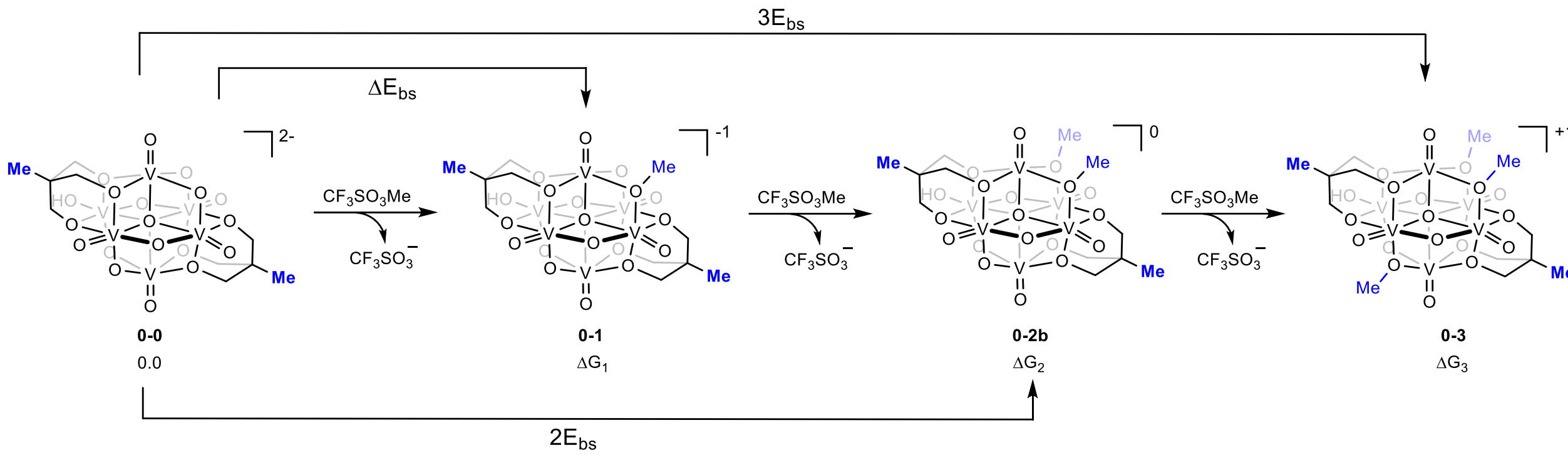


| Steps | ΔG$_1$-BS1 | ΔG$_1$-TZ | ΔG$_2$-BS1 | ΔG$_2$-TZ | ΔG$_3$-BS1 | ΔG$_3$-TZ |
|---|---|---|---|---|---|---|
| **0-0 to 0-3** | -14.3 | -20.2 | -17.4 | -28.9 | -9.5 | -26.8 |
| **1-0 to 1-3** | -31.9 | -37.3 | -45.0 | -55.6 | -52.8 | -69.2 |
| **2-0 to 2-3** | -47.5 | -52.3 | -79.9 | -89.9 | -95.1 | -110.3 |
| **3-0 to 3-3** | -64.4 | -68.9 | -111.3 | -120.3 | -149.4 | -163.4 |
| **4-0 to 4-3** | -84.0 | -87.9 | -157.7 | -165.8 | -207.5 | -220.3 |
| **5-0 to 5-3** | -96.6 | -99.7 | -179.6 | -186.4 | -254.6 | -265.6 |
| **6-0 to 6-3** | -106.5 | -108.7 | -202.3 | -205.9 | -288.4 | -295.6 |

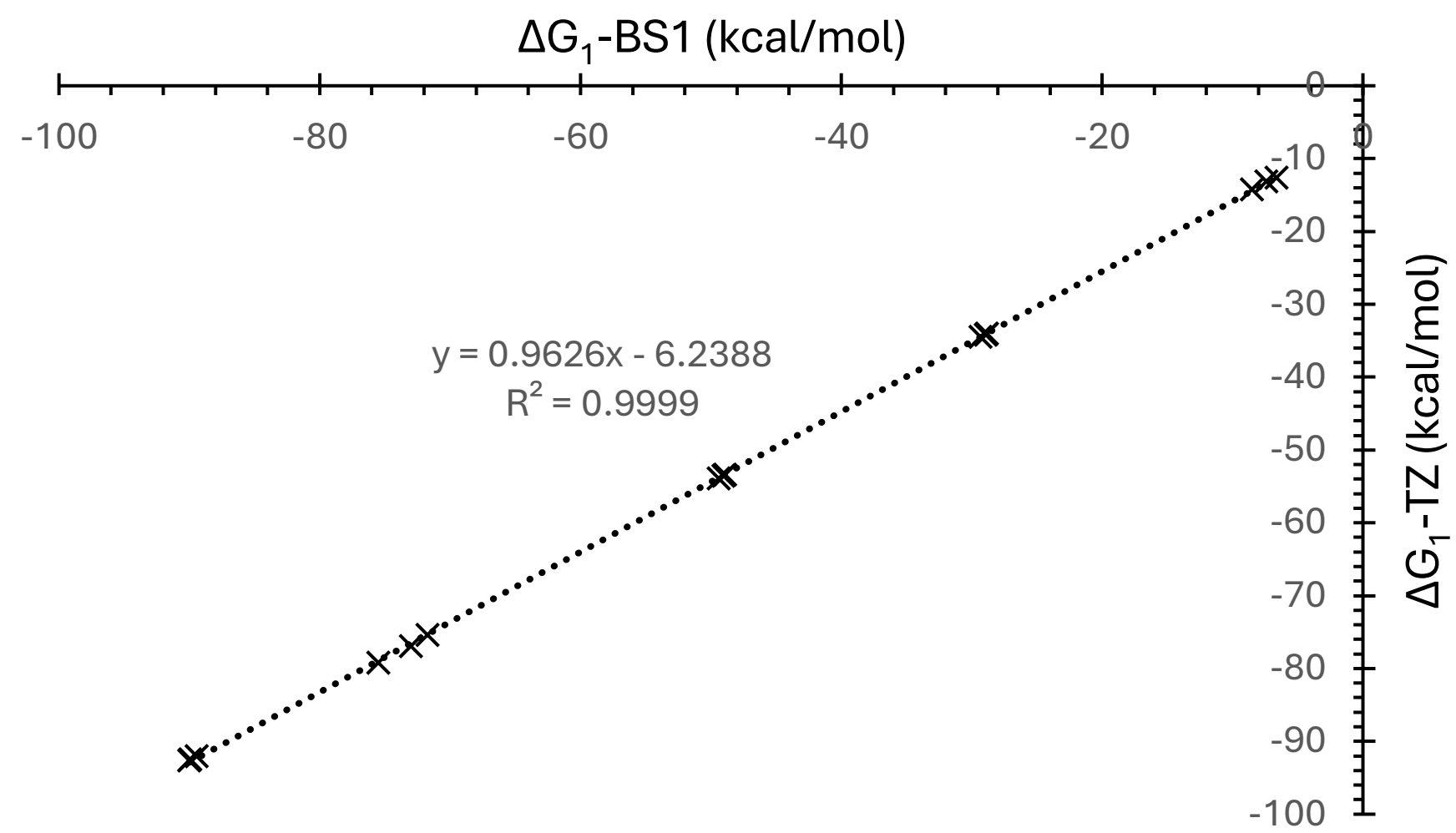


**Figure S18.** Correlation of the thermodynamic energies for the first methylation step in Table S5 between the SMD(THF)-PBE0-D4/BS1 and SMD(THF)-PBE0-D4/def2-TZVP//BS1 level of theory.

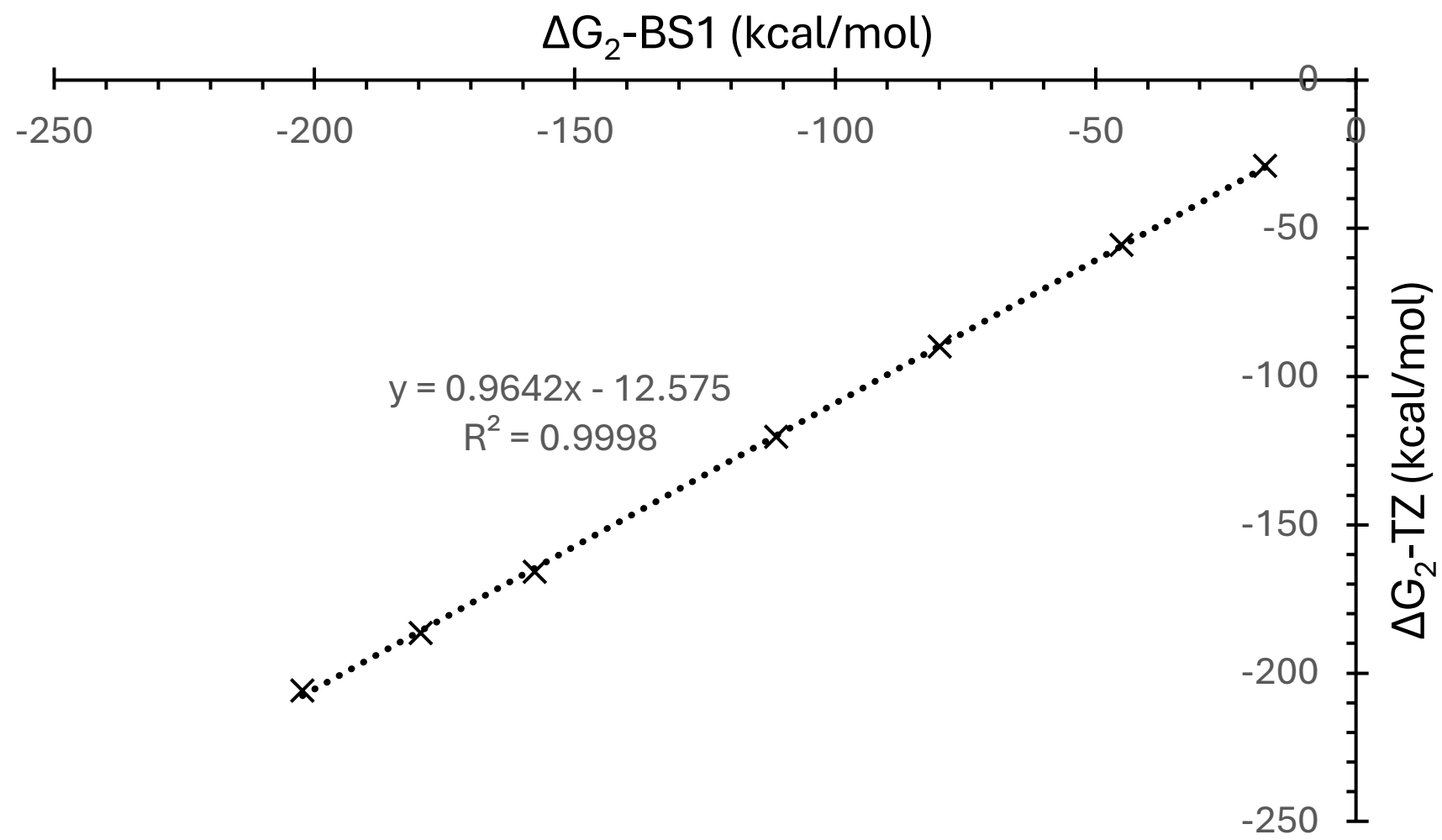


**Figure S19.** Correlation of the thermodynamic energies for the second methylation step in Table S5 between the SMD(THF)-PBE0-D4/BS1 and SMD(THF)-PBE0-D4/def2-TZVP//BS1 level of theory.

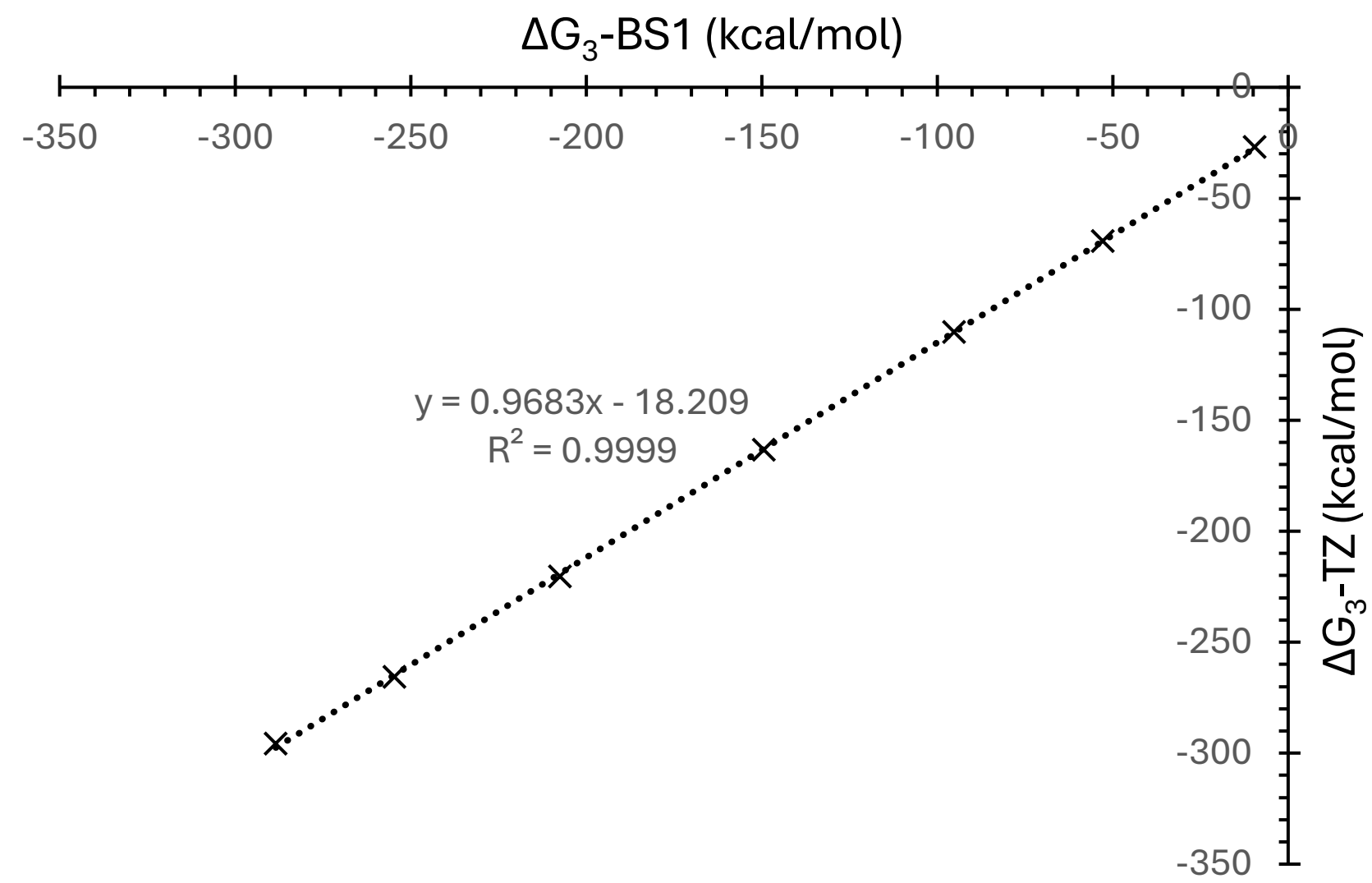


**Figure S20.** Correlation of the thermodynamic energies for the third methylation step in Table S5 between the SMD(THF)-PBE0-D4/BS1 and SMD(THF)-PBE0-D4/def2-TZVP//BS1 level of theory.

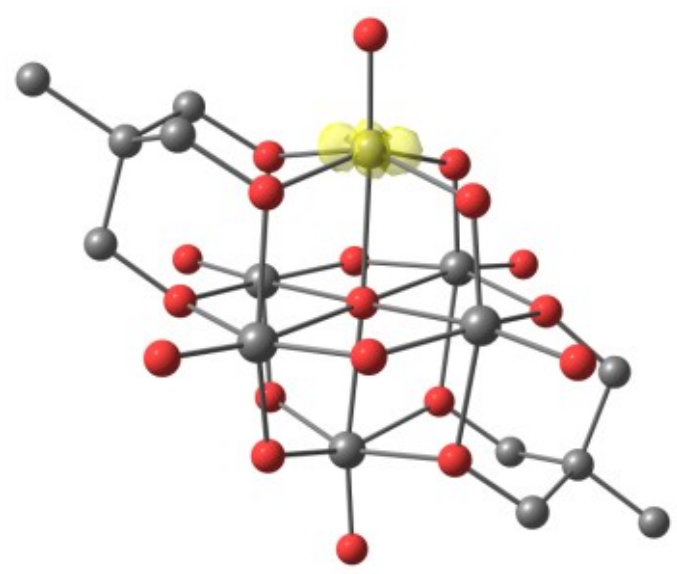

**1-0**

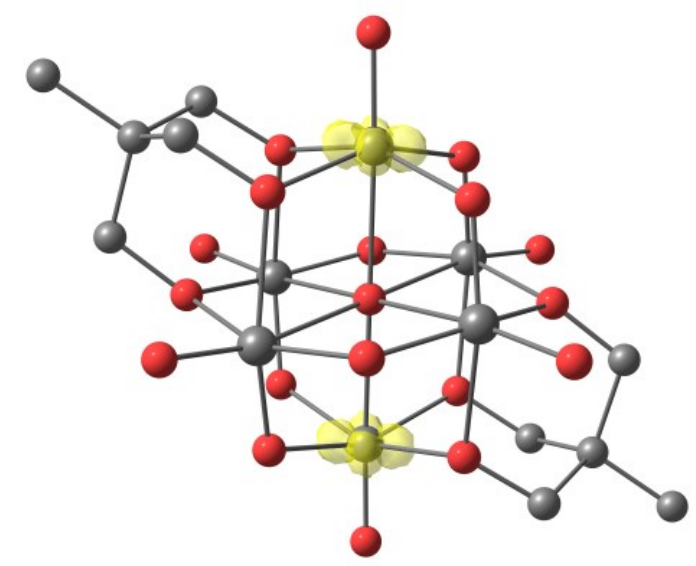

**2-0**

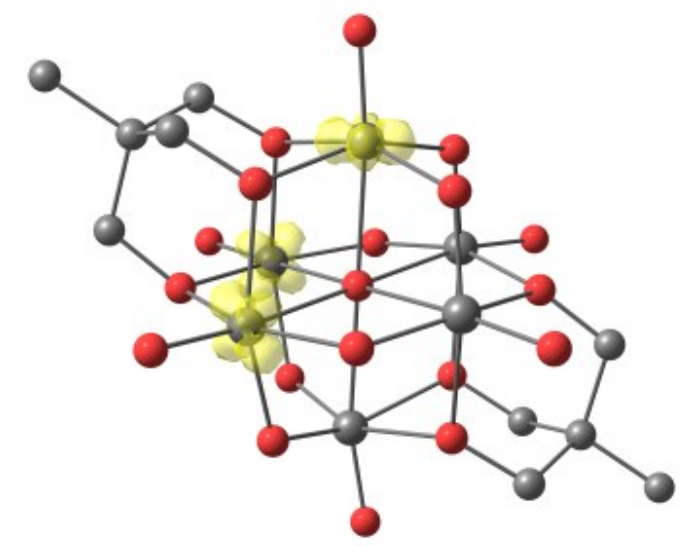

**3-0**

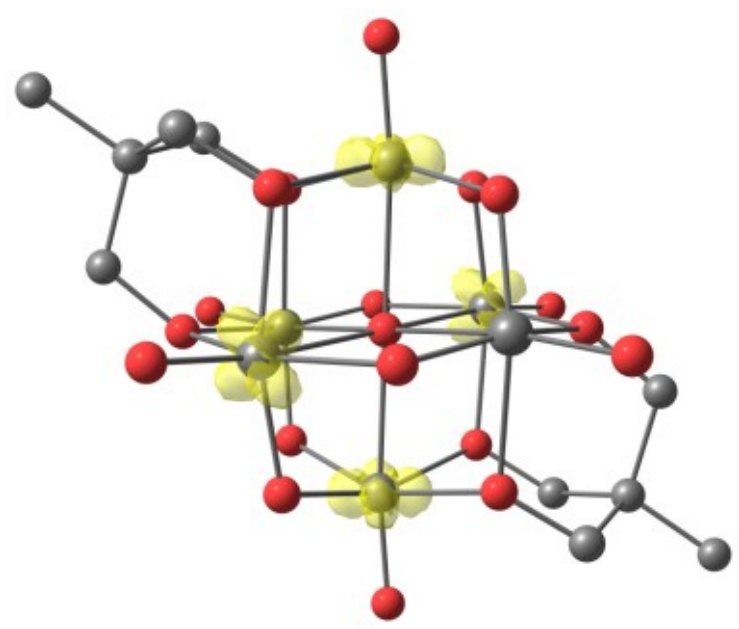

**4-0**

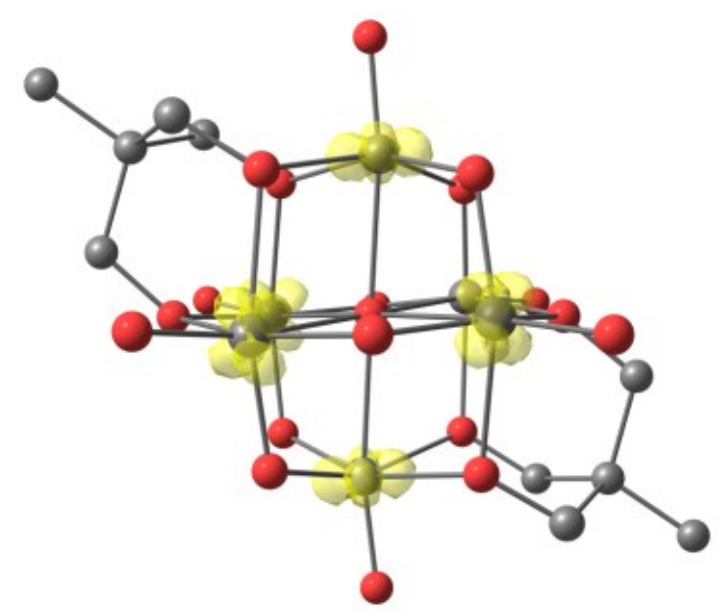

**5-0**

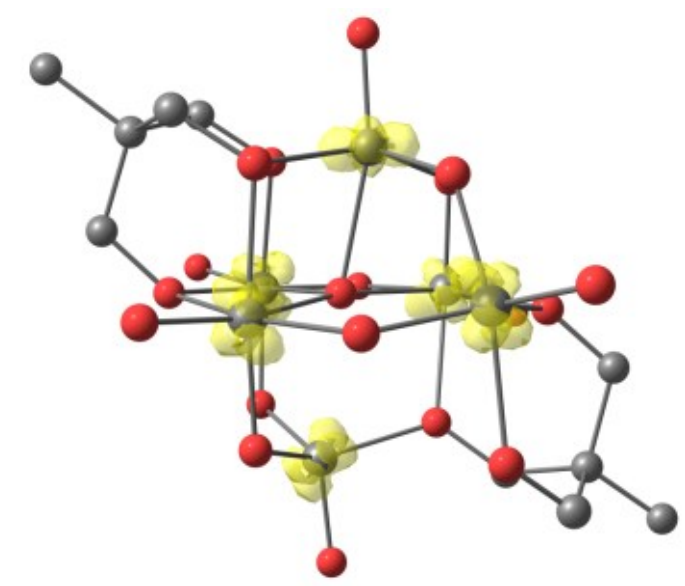

**6-0**

**Figure S21.** Spin Density Plots for Different Reduction States of the Nonmethylated TRIOL$^{Me}$ Cluster

**Table S6.** Computed results for transition state energies of selected methylation processes used to examine the effect of basis set choice. $\Delta\Delta G_1$–BS1 values were calculated at the SMD(THF)-PBE0-D4/BS1 level of theory, while $\Delta\Delta G_1$–TZ values were computed at the SMD(THF)-PBE0-D4/def2-TZVP//BS1 level of theory. The label ΔΔ indicates a Gibbs free energy for a single step rather than being referenced to the initial **0-0** species.

| **Steps** | **$\Delta\Delta G_1$-BS1** | **$\Delta\Delta G_1$-TZ** |
|---|---|---|
| **Before ion pair dissociation** | | |
| TS-0-01 | 25.2 | 24.8 |
| TS-1-01 | 20.2 | 20.1 |
| TS-2-01 | 15.1 | 15.0 |
| **After ion pair dissociation** | | |
| TS-0-12a | 14.7 | 8.5 |
| TS-0-12b | 14.8 | 8.8 |
| TS-0-12c | 13.9 | 7.9 |
| TS-1-12a | -7.4 | -13.1 |
| TS-1-12b | -8.5 | -14.2 |
| TS-1-12c | -6.6 | -12.6 |
| TS-2-12a | -28.8 | -34.0 |
| TS-2-12b | -28.9 | -34.2 |
| TS-2-12c | -29.3 | -34.5 |
| TS-0-2c-3a | 13.5 | 2.4 |
| TS-0-2b-3a | 13.3 | 2.5 |
| TS-0-2b-3b | 15.1 | 3.6 |
| TS-0-2a-3b | 14.7 | 3.5 |
| TS-0-2c-3b | 15.4 | 3.7 |
| TS-0-2c-3c | 13.8 | 2.1 |
| TS-1-2c-3a | -23.0 | -33.7 |
| TS-1-2b-3a | -23.4 | -33.8 |
| TS-1-2b-3b | -23.2 | -34.3 |
| TS-1-2a-3b | -20.1 | -30.9 |
| TS-1-2c-3b | -20.2 | -31.4 |
| TS-1-2c-3c | -21.3 | -32.4 |
| TS-2-2c-3a | -56.2 | -66.4 |
| TS-2-2b-3a | -57.8 | -67.8 |
| TS-2-2b-3b | -58.9 | -69.4 |
| TS-2-2a-3b | -57.0 | -66.8 |
| TS-2-2c-3b | -53.3 | -63.5 |
| TS-2-2c-3c | -56.4 | -67.0 |

**Table S7**. Computed results for selected reduction processes used to examine the effect of basis set choice. $\Delta G_1$–BS1 values were calculated at the SMD(THF)-PBE0-D4/BS1 level of theory, while $\Delta G_1$–TZ values were computed at the SMD(THF)-PBE0-D4/def2-TZVP//BS1 level of theory

| **X=Me, Y=Me** | **BS1-E(V)** | **TZ-ΔE(V)** |
|---|---|---|
| **0-0** to **1-0** | -0.32 | -0.24 |
| **1-0** to **2-0** | -1.12 | -1.06 |
| **2-0** to **3-0** | -1.83 | -1.83 |
| **3-0** to **4-0** | -2.97 | -2.98 |
| **4-0** to **5-0** | -3.81 | -3.85 |
| **5-0** to **6-0** | -4.29 | -4.45 |
| **X= $NO_2$, Y= $NO_2$** | **BS1-E(V)** | **TZ-ΔE(V)** |
| **0-0** to **1-0** | -0.75 | -0.68 |
| **1-0** to **2-0** | -1.49 | -1.46 |
| **2-0** to **3-0** | -2.71 | -2.72 |
| **3-0** to **4-0** | -2.99 | -3.35 |
| **4-0** to **5-0** | -3.03 | -3.40 |
| **5-0** to **6-0** | -3.65 | -3.73 |

**Table S8.** Computed results for the methylation processes used in microkinetic modeling presented in Figure 4. The reported values were calculated at the SMD(THF)-PBE0-D4/def2-TZVP//BS1 level of theory, and the TZ-ΔΔG values represent the activation barriers. The label ΔΔ indicates a Gibbs free energy for a single step rather than being referenced to the initial **0-0** species.

| Transition States | TZ-ΔG(kcal/mol) | TZ-ΔΔG(kcal/mol) |
|---|---|---|
| TS-2a-11 | 8.5 | 28.7 |
| TS-2b-11 | 8.8 | 29.0 |
| TS-2c-11 | 7.9 | 28.1 |
| TS-2a-22 | -13.2 | 24.1 |
| TS-2b-22 | -14.2 | 23.1 |
| TS-2c-22 | -12.6 | 24.7 |
| TS-2a-33 | -34.0 | 18.3 |
| TS-2b-33 | -34.2 | 18.1 |
| TS-2c-33 | -34.5 | 17.8 |
| TS-2a-3b-01 | 3.5 | 31.6 |
| TS-2b-3a-01 | 2.5 | 31.4 |
| TS-2b-3b-01 | 3.6 | 32.5 |
| TS-2c-3a-01 | 2.4 | 32.3 |
| TS-2c-3b-01 | 3.7 | 33.6 |
| TS-2c-3c-01 | 2.1 | 32.0 |
| TS-2a-3b-12 | -30.8 | 27.4 |
| TS-2b-3a-12 | -33.8 | 21.8 |
| TS-2b-3b-12 | -34.3 | 21.3 |
| TS-2c-3a-12 | -33.7 | 23.2 |
| TS-2c-3b-12 | -31.8 | 25.5 |
| TS-2c-3c-12 | -32.4 | 24.5 |
| TS-2a-3b-23 | -66.8 | 23.0 |
| TS-2b-3a-23 | -67.8 | 22.1 |
| TS-2b-3b-23 | -69.4 | 20.5 |
| TS-2c-3a-23 | -66.4 | 22.0 |
| TS-2c-3b-23 | -63.5 | 24.9 |
| TS-2c-3c-23 | -67.0 | 21.4 |
